# Separable Roles for a *Caenorhabditis elegans* RMI1 Homolog in Promoting and Antagonizing Meiotic Crossovers Ensure Faithful Chromosome Inheritance

**DOI:** 10.1371/journal.pbio.1002412

**Published:** 2016-03-24

**Authors:** Marlène Jagut, Patricia Hamminger, Alexander Woglar, Sophia Millonigg, Luis Paulin, Martin Mikl, Maria Rosaria Dello Stritto, Lois Tang, Cornelia Habacher, Angela Tam, Miguel Gallach, Arndt von Haeseler, Anne M. Villeneuve, Verena Jantsch

**Affiliations:** 1 Department of Chromosome Biology, Max F. Perutz Laboratories, Vienna Bio Center, University of Vienna, Vienna, Austria; 2 Departments of Developmental Biology and Genetics, Stanford University School of Medicine, Stanford, California, United States of America; 3 Center for Integrative Bioinformatics Vienna (CIBIV), Max F. Perutz Laboratories, Vienna Bio Center, University of Vienna and Medical University of Vienna, Vienna, Austria; 4 Bioinformatics and Computational Biology, Faculty of Computer Science, University of Vienna, Austria; National Cancer Institute, UNITED STATES

## Abstract

During the first meiotic division, crossovers (COs) between homologous chromosomes ensure their correct segregation. COs are produced by homologous recombination (HR)-mediated repair of programmed DNA double strand breaks (DSBs). As more DSBs are induced than COs, mechanisms are required to establish a regulated number of COs and to repair remaining intermediates as non-crossovers (NCOs). We show that the *Caenorhabditis elegans* RMI1 homolog-1 (RMH-1) functions during meiosis to promote both CO and NCO HR at appropriate chromosomal sites. RMH-1 accumulates at CO sites, dependent on known pro-CO factors, and acts to promote CO designation and enforce the CO outcome of HR-intermediate resolution. RMH-1 also localizes at NCO sites and functions in parallel with SMC-5 to antagonize excess HR-based connections between chromosomes. Moreover, RMH-1 also has a major role in channeling DSBs into an NCO HR outcome near the centers of chromosomes, thereby ensuring that COs form predominantly at off-center positions.

## Abstract 2

A nematode homolog of the conserved DNA repair factor RMI1 plays multiple genetically separable roles that together ensure the faithful inheritance of intact genomes during sexual reproduction.

## Author Summary

During meiosis, faithful separation of chromosomes into gametes is essential for fertility and healthy progeny. During the first meiotic division, crossovers (CO) between parental homologs ensure their correct segregation. Programmed DNA double strand breaks (DSBs) and resection steps generate single-stranded overhangs that invade a sister chromatid of the homolog to initiate homologous recombination. This culminates in the generation of a DNA double Holliday junction (dHJ). This can be acted upon by resolvases to produce CO and non-crossover (NCO) products, depending on where the resolvases cut the DNA. Alternatively, NCOs can also be produced by decatenation via the RecQ helicase–topoisomeraseIII–Rmi1 (RTR) complex. The mammalian RTR contains a topoisomerase, Bloom’s helicase, and RMI1/2 scaffolding components. It disassembles dHJs in vitro and contributes the major NCO activity in mitosis. Here, we provide evidence that the *Caenorhabditis elegans* RMH-1 functions in distinct complexes during meiosis to produce both COs and NCOs in an in vivo animal model system. Strikingly, RMH-1 spatially regulates the distribution of COs on chromosomes, demonstrating that the RTR complex can act locally within specific chromosome domains.

## Introduction

During meiosis, the accurate segregation of chromosomes relies on the formation of crossovers (COs) and sister chromatid cohesion [1]. COs are produced by homologous recombination (HR)-mediated repair of programmed DNA double strand breaks (DSBs). In *Caenorhabditis elegans*, the holocentric chromosome pairs usually undergo a single off-center CO, resulting in a cruciform and asymmetric bivalent [2,3]. This is important to define the organization of kinetochore proteins, motor proteins, protein kinases, and the domains of cohesion release, which is necessary for the accurate segregation of homologs during anaphase I [4].

During prophase I, several mechanisms guarantee the formation of COs as well as the repair of the additional recombination intermediates as non-crossovers (NCOs).

First, to ensure CO formation, programmed DSBs are induced in excess compared to the actual number of COs, e.g., [5–7]. Second, a subset of the early recombination intermediates are stabilized and protected by pro-CO factors [8–10]. CO maturation is supported by the MutSγ complex (MSH-4/5), sumo/ubiquitin ligases ZHP-3/RNF212, and HEI10 and the cyclin-related protein COSA-1/CNTD1 [11–16]. In addition to efficient CO designation, it is essential that the resolution of joint molecules (JM) at CO-designated sites is biased toward the CO outcome. JMs result from second-strand capture and DNA synthesis after strand invasion [17]. Depending on where the cut is made by structure-specific endonucleases (resolvases), CO or NCO products can be generated [18–21]. In *C*. *elegans*, at least two parallel pathways of a redundant resolvase system have been defined (MUS-81/SLX-1 and XPF-1/HIM-6) [22–24]. It is unclear how the bias in resolution toward either a CO or NCO is achieved.

In yeast, the bulk of DSBs not destined to become COs are processed by synthesis-dependent strand annealing (SDSA) at an early stage [25]. In *C*. *elegans*, a similar activity is attributed to RTEL-1 by D-loop displacement after strand invasion [26,27]. NCOs can also be generated by the activity of the RTR complex, composed of a RecQ helicase (Bloom syndrome protein [BLM]), topoisomerase IIIα (TOP3), and the RecQ-mediated genome instability 1 and 2 (RMI1,2) scaffolding proteins [28–30]. This complex provides a major NCO activity during mitosis. Importantly, patients with mutated BLM helicase display genomic instability and a cancer predisposition, and they exhibit a cellular phenotype of elevated COs between sister chromatids, which potentially drives cancer by loss of heterozygosity [31,32]. In vitro studies have shown that the RTR complex dismantles double Holliday junctions (dHJs). A branch migration step mediated by the RecQ helicase brings the two dHJs in close proximity to allow the topoisomerase to unhook the two DNA strands by decatenation, stimulated by the scaffolding proteins RMI1/2 [33].

RMI1 was discovered as a protein associated with BLM containing protein complexes [34]. It colocalizes with recombination foci containing BLM helicase [35]. RMI1 depletion leads to destabilization of BLM and topoisomerase foci. Moreover, its depletion phenocopies the BLM mutant phenotype [35]. Furthermore, RMI1 directly interacts with both BLM helicase and TOP3, shown with pulldown experiments using recombinant protein [36]. Human RMI1 protein contains two oligonucleotide/oligosaccharide-binding (OB) fold domains, in which the N-terminal OB fold binds both the helicase and the topoisomerase [37]. Structural and biochemical studies established that RMI1 cooperates with the topoisomerase in the strand passage reaction during decatenation. The topoisomerase-generated nick allows strand passage of the other strand, in which RMI1 plays a role in the regulation of opening and closure of the “gate” [38,39].

An “anti-CO” role has been described for BLM orthologs in yeast, plants, mice, and *Drosophila* in meiosis [25,40–45]. A recent study showed that, in *C*. *elegans*, *him-6* is required to eliminate persistent MutSγ-independent recombination intermediates when an excess is induced by irradiation [46]. Similar “anti-CO” functions have been attributed to other members of the complex: Rmi1 and/or Top3 in yeast and *Arabidopsis* [47–52]. In yeast, Top3 and Rmi1 cooperate with Sgs1 to prevent aberrant joint molecule (JM) accumulation, but they also act later, without Sgs1, to allow chromosome separation [51,52].

Despite the widely conserved anti-CO function of the RTR complex, several studies suggest that it could also contribute a pro-CO activity; however, a direct involvement has not been demonstrated. In *C*. *elegans*, *him-6* (the BLM ortholog) is required to ensure that CO-designated recombination intermediates mature into interhomolog COs [46,53,54]. In yeast, Sgs1 has been found colocalizing with the pro-CO factor Zip3 [40]. Nevertheless, in *sgs1* mutants, the recombination rate is increased rather than decreased, as one would expect if it favored CO formation. More recently, the Sgs1/BLM helicase has been proposed to have an early role in promoting COs, acting to disassemble unprotected strand invasion intermediates to channel them back into CO pathways [41,44,45,55,56]. Furthermore, in a situation in which JM resolution is blocked (*mms4 slx4 yen1*), Sgs1 can promote CO formation. Here, *sgs1* might generate JMs with a certain configuration that could be recognized by the major yeast CO pathway Exo1-MutLγ [55].

In this study, we present the role of *C*. *elegans* RMH-1. We show that RMH-1 and HIM-6 localize at numerous recombination intermediates that will become either a CO or NCO. Consistent with its localization, RMH-1 plays genetically separable and antagonistic roles during meiosis. On one hand, *rmh-1* ensures CO formation at two levels: by promoting CO designation and by protecting CO-designated sites and promoting their maturation into chiasmata. Remarkably, RMH-1 and HIM-6 localize as closely juxtaposed doublets, suggesting that they may flank the two junctions of a dHJ CO intermediate. In addition, *rmh-1* prevents illegitimate connections between homologs redundantly with *smc-5*, an activity known from yeast meiosis to prevent multi-JMs [57–59]. Moreover, RMH-1 anti-CO activity is required to inhibit CO formation at chromosome centers, which would facilitate correct segregation of chromosomes.

## Results

### Identification of *rmh-1* Mutants

**Fig 1:**
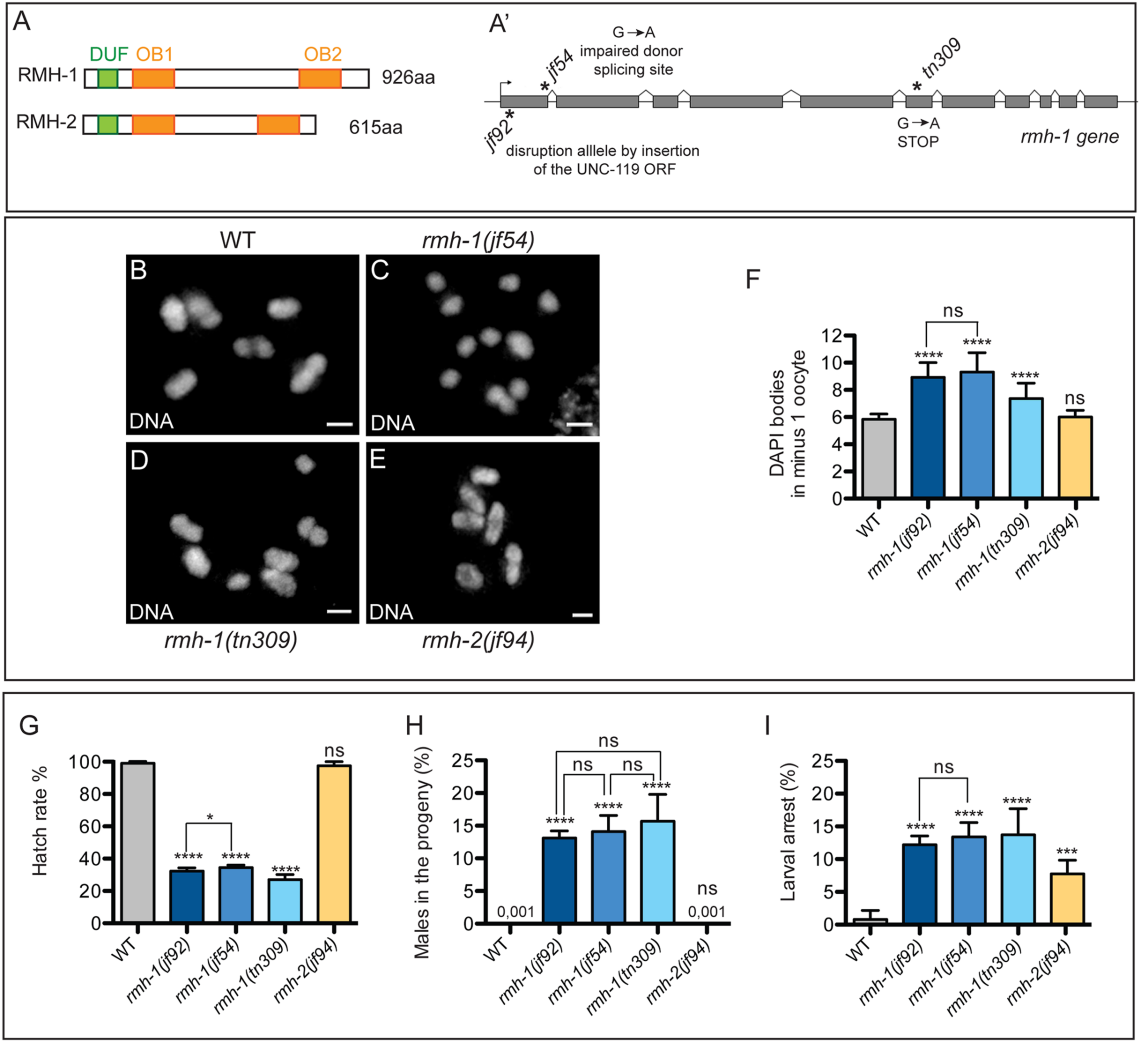
RMH-1 (but not RMH-2) contributes to reliable chiasma formation and chromosome segregation in meiosis. (A) Schematics of RMH-1 and RMH-2. (A′) Location of the three *rmh-1* mutations. In the *jf92* allele, the coding sequence was disrupted after the START codon by the insertion of the *unc-119* gene by the CRISPR technology. In the *jf54* allele, the G-to-A transition affects the first nucleotide of intron 1 and therefore, destroys the splice donor site of the preceding exon 1. qRT-PCR revealed the presence of different splicing variants (see S1 Fig). In the *tn309* allele, the G-to-A transition introduces a premature STOP codon at position aa 640, leading to the deletion of the OB2 domain (for more details, see the Experimental Procedures section). (B–E) Oocyte nuclei at the diakinesis stage of meiotic prophase. Each image shows the complete set of DAPI-stained chromosomes from a single nucleus. The wild-type (WT) and *rmh-2(jf94)* nuclei contain six bivalents (homolog pairs connected by chiasmata), whereas the *rmh-1* mutant nuclei contain a mixture of bivalents and univalents. (F) Quantification of the average number of DAPI-positive structures in diakinesis oocytes in the -1 position. WT *n* = 25, *rmh-1(jf92) n* = 21, *rmh-1(jf54) n* = 36, *rmh-1(tn309) n* = 30, and *rmh-2(jf94) n* = 18. (G–I) Quantification of embryonic hatch rates (G). Frequencies of male offspring (H) and larval arrest (I) among the progeny of WT and *rmh-1* and *rmh-2* mutant worms (*n* = 35–45 hermaphrodites per genotype). Data for F–I are represented as mean +/- SD; ns stands for not significant, differences are highlighted with stars (* *p* < 0.05, ** *p* < 0.01, *** *p* < 0.001. and **** *p* < 0.0001). Scale bars, 2 μm.

The *C*. *elegans* genome encodes two RMI1 homologs: M01E11.3 and T07C12.12, named RMI1 homolog-1 (RMH-1) and -2 (RMH-2), respectively. These proteins contain three domains characteristic of RMI1 orthologs: an N-terminal domain of unknown function (DUF)1767 and two OB-fold domains (Fig 1A and S1A Fig). Point mutations in the recessive alleles *rmh-1(jf54*) and *rmh-1(tn309)* were isolated based on defects in meiotic chromosome segregation and embryonic lethality [60–62]. The mutations lead to protein truncations after the DUF1767 or OB1 domains, respectively (Fig 1A′ and S1B–S1D Fig). In *rmh-1(jf92)* and *rmh-2(jf94)*, the ORFs were disrupted right after the start codon (see the Experimental Procedures section).

### RMH-1 (But Not RMH-2) Is Required for Robust Chiasma Formation and Chromosome Segregation in Meiosis

To establish the respective requirements of *rmh-1* and *rmh-2*, we examined the mutants for embryonic lethality, larval arrest, and a high incidence of males (Him) phenotype among the progeny. An increased incidence of males in the progeny indicates X chromosome nondisjunction during meiosis, as *C*. *elegans* hermaphrodites have the genotype X/X and males X/O. The combination of increased embryonic lethality with a Him phenotype further suggests the occurrence of aneuploid eggs arising from meiotic missegregation of autosomes [63]. Increased larval arrest is indicative of somatic defects.

Analysis of *rmh-1* mutants revealed the cohort of phenotypes characteristic of a defect in meiotic recombination, as described above (Fig 1G and 1H). Second, DAPI staining of oocytes at diakinesis, the last stage of meiotic prophase, revealed that a fraction of chromosome pairs was not connected by chiasmata in *rmh-1* mutants. Whereas in the wild type, six bivalents (chromosome pairs connected by chiasmata) can be seen in diakinesis oocytes, *rmh-1* mutant alleles showed an increase of DAPI-stained bodies, reflecting a mixture of bivalents and univalents (achiasmate chromosomes) (Fig 1B–1E). In the -1 oocyte (the most mature oocyte, next to the spermatheca, the oocyte prior to fertilization), we observed on average 8.9 ± 1.0 DAPI bodies in *rmh-1(jf92)* and 9.3 ± 1.4 in *rmh-1(jf54)* (Fig 1F). As the *jf54* allele behaved identically to the *jf92* insertion allele with regards to the hatch rate and Him and diakinesis phenotypes, and as we were unable to detect GFP::RMH-1 containing the *jf54* mutation (S2B Fig), we infer that *rmh-1(jf54)* behaves like a null allele. Interestingly, fewer univalents were observed in *rmh-1(tn309)* (7.4 ± 1.1 DAPI bodies), suggesting that RMH-1 function is differently affected by this allele (Fig 1F). To confirm the presence of univalents in *rmh-1* mutants (and not fragments due to absence of DNA repair), we measured the average volumes of DAPI bodies in diakinesis (S3A and S3B Fig and S1 Text) and confirmed both the presence of univalents and the absence of DNA fragments.

In contrast to the *rmh-1* mutants, the *rmh-2(jf94)* single mutant did not exhibit any meiotic phenotypes. However, all *rmh* mutants showed increased levels of larval arrest (around 12% for *rmh-1* mutants and 7% for *rmh-2*) (Fig 1I). Furthermore, the double mutant *rmh-1(jf54); rmh-2(jf94)* was inviable, suggesting a redundant function of the two genes in somatic tissue.

**Fig 2:**
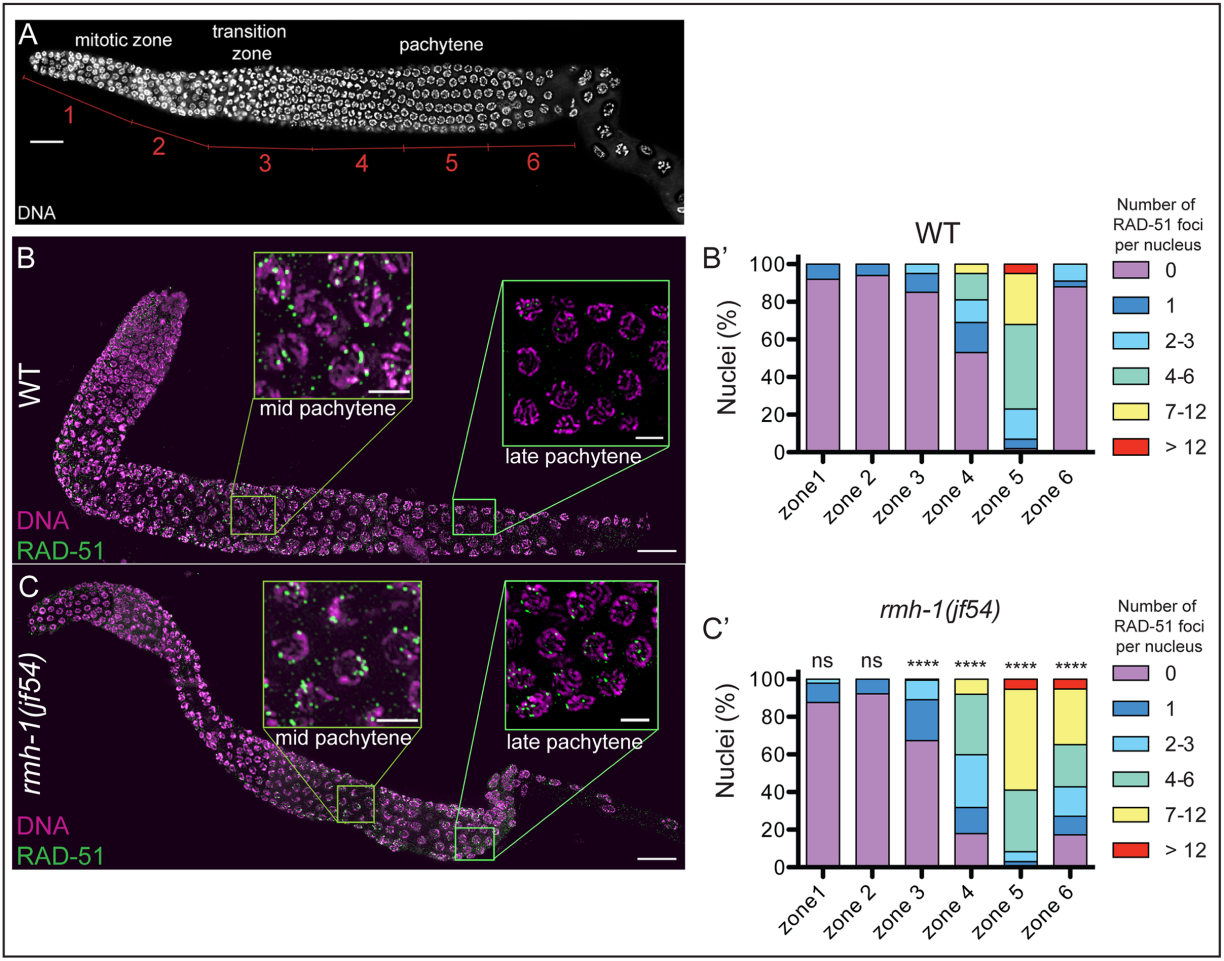
DNA double strand break repair is delayed in *rmh-1* mutant. (B–C) Immunostaining for DNA strand exchange protein RAD-51, with insets for mid pachytene (MP) and late pachytene (LP) nuclei. (B) Almost no RAD-51 foci are detected in LP nuclei in wild-type (WT). (C) Abundant RAD-51 foci are still detected in LP nuclei in the *rmh-1(jf54)* mutant, but disappear in diplotene. (B′,C′) Quantification of the numbers of RAD-51 foci per nucleus. For quantification, gonads were divided into six equal zones (A). In both WT and *rmh-1(jf54)*, zones 1 and 2 correspond to the mitotic zone; zone 3 to transition zone and early pachytene; zones 4 to 5 to pachytene. Analysis of *n* = 124–231 nuclei per zone for each genotype. Distribution of RAD-51 foci is significantly different between WT and mutant for the zones 3 to 6 (Mann Whitney test, **** *p* < 0.0001). Scale bar = 20 μm for gonads and 5 μm for insets.

To better understand which meiotic processes are impaired in *rmh-1(jf54)*, we analyzed chromosome pairing, synapsis, and induction and repair of DSBs. No defects in pairing and synapsis were observed (S4 Fig). To study the induction and processing of DSBs, we assessed the appearance and disappearance of foci of the strand exchange protein RAD-51 (Fig 2) [64,65]. RAD-51 foci appeared with normal timing in the *rmh-1(jf54)* mutant, but accumulated at higher levels than in wild type and persisted at elevated levels through late pachytene. However, RAD-51 foci disappeared in diplotene, suggesting that repair of DSBs was delayed but did eventually occur. We also found that *rmh-1*-specific chromosomal defects depended on the induction of meiotic DSBs. In summary, meiotic recombination is aberrant in *rmh-1* mutants.

### Dynamic Localization of RMH-1 in Distinct Foci during Pachytene Progression

**Fig 3:**
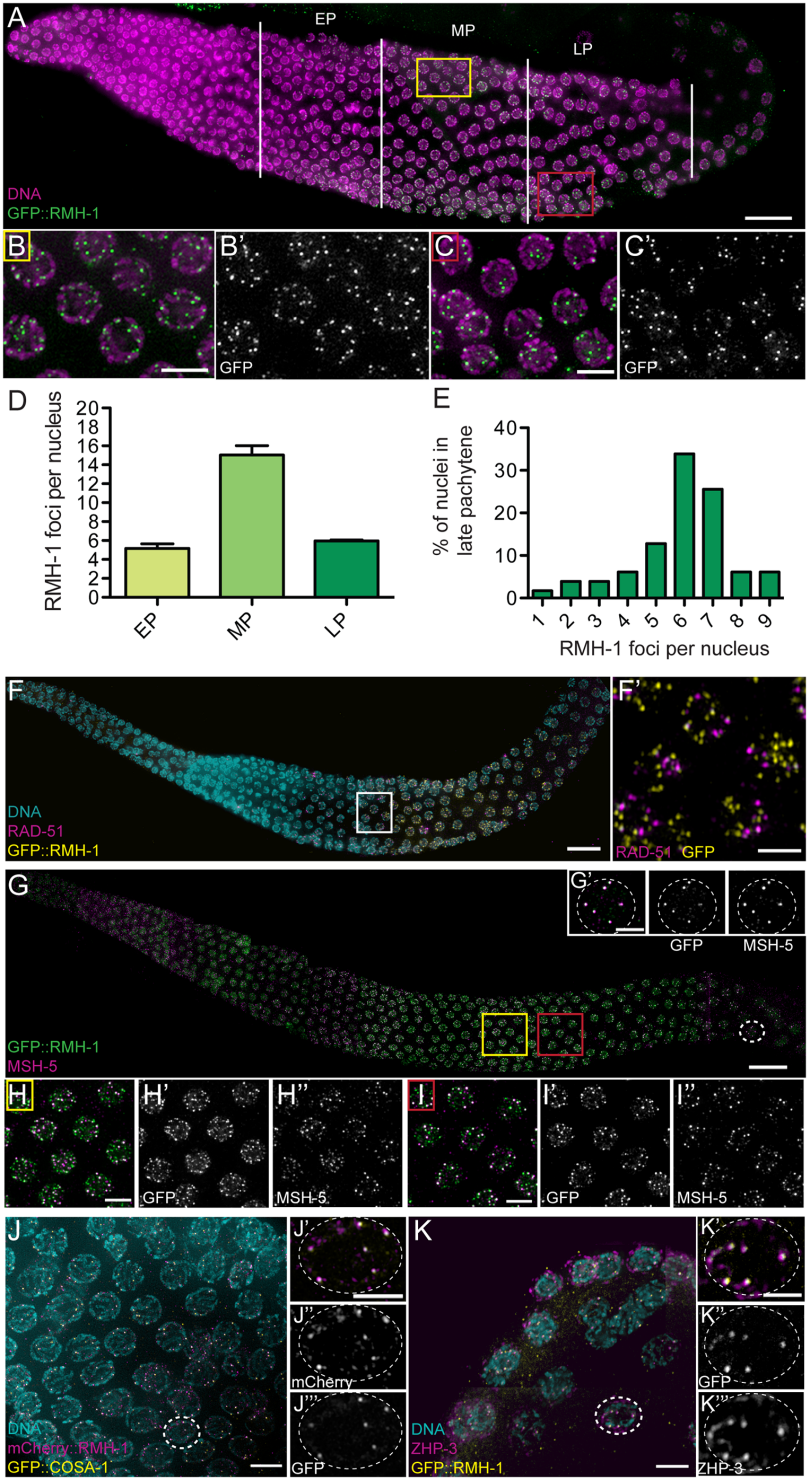
Dynamic localization of RMH-1 to distinct foci in pachytene. (A) In early pachytene (EP), GFP::RMH-1 is diffuse and few foci are detected on DNA. In mid pachytene (MP), high numbers of foci (up to 25 per nucleus) are observed (yellow square and B and B′). In late pachytene (LP), GFP::RMH-1 concentrates into bright foci, on average six per nucleus (red square and C and C′). (D) Quantification of the average number of RMH-1 foci per nucleus in EP, MP, and LP (*n* = 123 nuclei for EP, *n* = 205 for MP, and *n* = 180 for LP). Data are represented as mean +/- SEM. (E) Histogram showing the percentage of late pachytene nuclei containing a certain number of RMH-1 foci per nucleus (one to nine) (*n* = 180 nuclei). (F) Staining for RAD-51 and GFP::RMH-1. RMH-1 localization starts after and persists longer than the RAD-51 positive zone. (F′) GFP::RMH-1 and RAD-51 mark different recombination intermediates, as they do not colocalize. (G–I) Staining for MSH-5 and GFP::RMH-1. (H–H′′) RMH-1 and MSH-5 partially colocalize in MP (yellow square). (I–I′′) Both proteins become enriched at brighter foci as pachytene progresses (red square). (J) Staining for COSA-1::GFP and mCherry::RMH-1. (K) Staining for ZHP-3 and GFP::RMH-1. In LP, RMH-1 colocalizes at CO sites with MSH-5 (G′), COSA-1 (J′–J′′′) and ZHP-3 (K′–K′′′). Scale bar = 20 μm for gonads, 5 μm for pachytene nuclei insets, and 2.5 μm for single nucleus.

To assess RMH-1 localization in the gonad, we expressed GFP::RMH-1 using its endogenous regulatory elements as a functional fusion protein. Indeed, GFP::RMH-1 is capable of rescuing embryonic lethality and the Him phenotype when expressed in the null allele *jf92* (S5A and S5B Fig). GFP::RMH-1 was found in foci throughout pachytene (Fig 3A–3C). In early pachytene, RMH-1 was first detected as a nucleoplasmic haze, and faint foci progressively appeared on chromosomes. During mid pachytene, RMH-1 foci increased in number and intensity. Mid pachytene nuclei contained, on average, 15.2 foci ± 3.6, ranging from ten to 25, and, in late pachytene, the number of RMH-1 foci drastically reduced to 5.9 ± 1.7 (Fig 3D). In late pachytene, the number of foci ranged from one to nine foci; however, the majority of nuclei contained six or seven foci (Fig 3E). (This distribution is broader than the distribution of COSA-1 foci, visible as six signals in late pachytene. However, the mutant phenotype of *cosa-1* already suggests that it acts before the protein can be observed in the six prominent foci. In addition, we believe RMH-1 marks a stage of recombination, through which all mature CO intermediates have to pass).

Importantly, RMH-1 foci seem to increase in signal intensity (about 1.8-fold) between mid and late pachytene. This suggested that the protein might accumulate over time.

RMH-1 localization in foci in pachytene is reminiscent of localization of markers of ongoing recombination events such as RAD-51 and CO-promoting factors MSH-5, ZHP-3, or COSA-1 [12,64–66]. Moreover, RMH-1 localization depends on meiotic DSBs, since foci were absent in a *spo-11* mutant (S2A Fig). Co-immunostaining experiments indicated that RAD-51 foci appear and disappear earlier than RMH-1 (S6 Fig). RAD-51 foci appear in transition zone, peak in mid pachytene, and disappear in late pachytene. GFP::RMH-1 foci appear in early pachytene, peak in mid pachytene, and disappear in diplotene. Moreover, when RAD-51 and RMH-1 are present in the same nucleus, they do not colocalize (only 4.0 +/- 4.0% RMH-1 foci coincide with RAD-51) (Fig 3F and 3F′). This suggests that RAD-51 and RMH-1 mark different recombination intermediates and that RMH-1 may act after RAD-51 removal.

**Fig 4:**
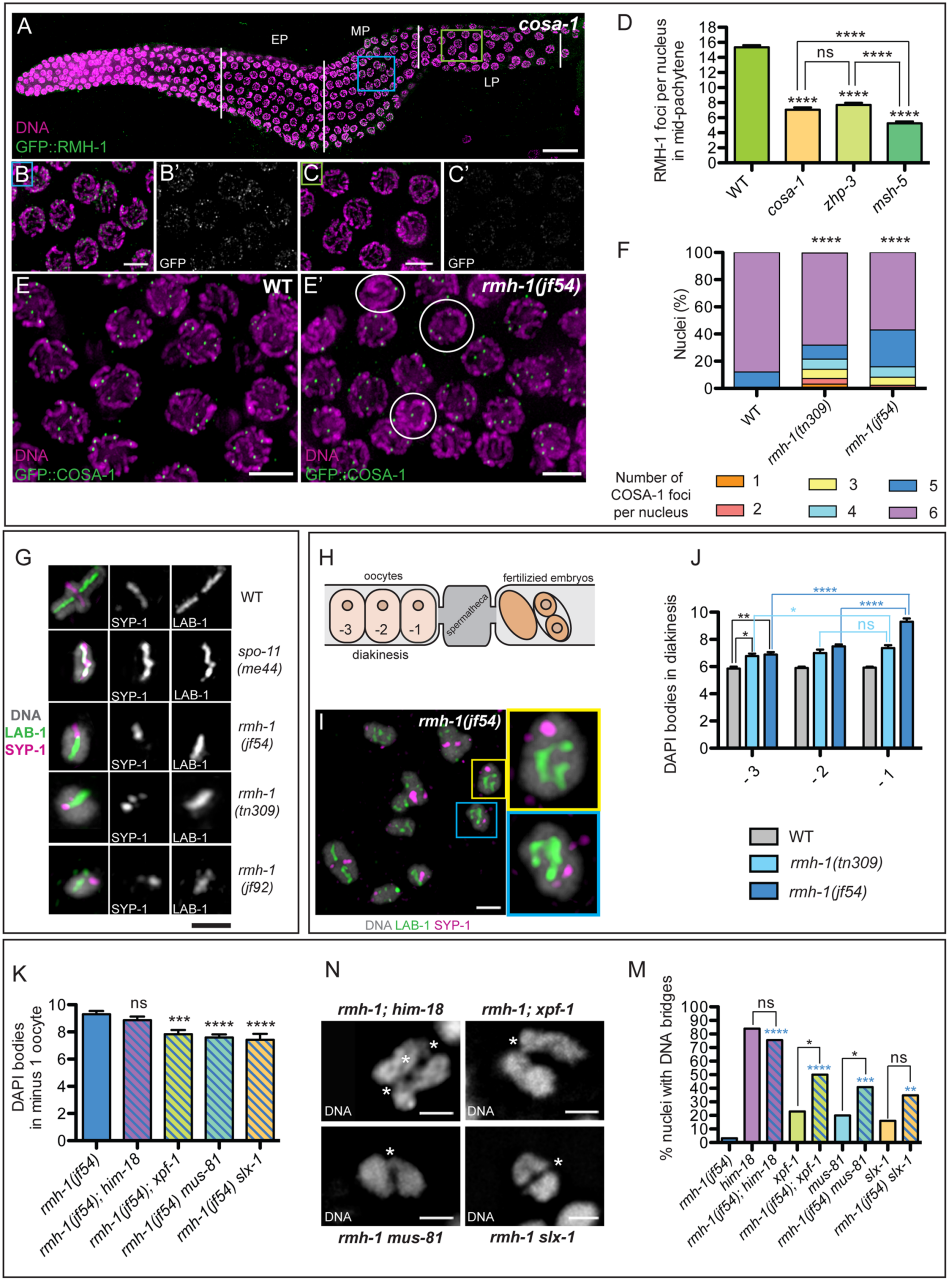
RMH-1 in the context of CO formation. (A–C) RMH-1 localization in a *cosa-1* mutant. In mid pachytene (MP), RMH-1 foci are less abundant and smaller than in wild-type (WT, blue square, B and B′) and are barely detectable in late pachytene (LP) (green square, C and C′). (D) Quantification of average RMH-1 foci per nucleus in MP in the WT (*n* = 205) and *cosa-1* (*n* = 214), *zhp-3* (*n* = 179), and *msh-5* (*n* = 137) mutants. In three CO-defective mutants, foci numbers were significantly reduced (*p* < 0.0001). Data are represented as mean +/- SEM. (E) In WT, most LP nuclei exhibit six COSA-1::GFP foci. (E′) In *rmh-1(jf54)*, nuclei with fewer than five COSA-1 foci (white circles) are observed. (F) Stacked bar graph showing the percentage of late pachytene nuclei containing a defined number of COSA-1 foci either in WT (*n* = 428) or in *rmh-1(jf54)* (*n* = 291) and *rmh-1(tn309)* (*n* = 329) mutants. Distribution of COSA-1 foci is significantly different between WT and both mutants (Mann Whitney test, **** *p* < 0.0001). (G) Images of individual bivalents (in WT) or univalents (in mutants) from diakinesis-stage oocytes, stained for markers that concentrate on the short arms (SYP-1) and long arms (LAB-1) of the cross-shaped bivalents in WT, reflecting differentiation of chromosome axis composition that occurs in response to nascent CO events. In *spo-11(me44)*, SYP-1 and LAB-1 domains overlap on univalents. In contrast, some univalents in *rmh-1* mutants exhibit spatially differentiated SYP-1 and LAB-1 domains, suggesting that CO sites were designated but did not mature into chiasmata. The example of *rmh-1(tn309)* shows that the mutual exclusion of LAB-1 and SYP-1 can be interrupted. (J) Quantification of DAPI positive structures in the -3, -2, and -1 oocytes (*n* = 31 for WT, *n* = 86 for *rmh-1(tn309)*, and *n* = 91 for *rmh-1(jf54)*), as shown in the scheme, in which the position is defined relative to the spermatheca (H). Homologs progressively dissociate in *rmh-1*. Data are represented as mean +/- SEM with ns for not significant, * *p* < 0.05, ** *p* < 0.01, and **** *p* <0.0001. (I) *rmh-1(jf54)* diakinesis nucleus, showing a pair of univalents (yellow and blue squares). (K) Quantification of DAPI positive structures in the -1 oocyte. Reduced DAPI bodies in the double *rmh-1(jf54); xpf-1* (*p* < 0.05), *rmh-1(jf54) mus-81* (*p* < 0.001), or *rmh-1(jf54) slx-1* (*p* < 0.01) but not *rmh-1(jf54); him-18* (*n* = 15–36 nuclei per genotype). Data are represented as mean +/- SEM with ns for not significant, * *p* < 0.05, ** *p* < 0.01, and **** *p* < 0.0001. (N) DNA bridges in *rmh-1(jf54); him-18*, *rmh-1(jf54); xpf-1*, *rmh-1(jf54) mus-81; and rmh-1(jf54) slx-1*. (M) Quantification of diakinesis nuclei (-2 and -1) containing at least one DNA bridge between two univalents (*n* = 30–45 nuclei per genotype). Blue stars represent differences to *rmh-1(jf54)*. Data are represented with **** for *p* < 0.001, * for *p* < 0.05, and ns for not significant (Chi^2^ test). Scale bars = 20 μm for gonads, 5 μm for nuclei, and 2 μm for bivalents or univalents.

In contrast with the lack of colocalization with RAD-51 foci, the six bright RMH-1 foci in late pachytene nuclei do coincide with the localization of pro-CO factors MSH-5, ZHP-3, and COSA-1 at the CO sites (90.1 +/- 10.7% RMH-1 foci colocalize with COSA-1 and MSH-5) (Fig 3G, 3J and 3K) [12,16,66]. Interestingly, RMH-1 foci disappear before COSA-1 foci, which persist until diplotene (S6 Fig). RMH-1 foci also partially colocalize with the earlier MSH-5 foci present during mid pachytene; the brighter foci of each protein tend to colocalize, while the fainter ones do not (Fig 3G–3I). Furthermore, we found that late-pachytene RMH-1 foci are severely reduced (to one focus on average) in *msh-5*, *zhp-3*, and *cosa-1* mutants, which fail to form CO-designated recombination intermediates. RMH-1 foci in mid pachytene are also less abundant and smaller in these mutants (Fig 4A–4D, S7D and S7E Fig), suggesting that *msh-5*, *zhp-3*, and *cosa-1* also contribute to stable localization of RMH-1 during earlier stages. The dynamic localization of RMH-1 through pachytene suggests that it may function at both CO and NCO repair events.

The mid pachytene number of RMH-1 foci, at least twice the number of eventual COs, is consistent with RMH-1 localizing both at CO sites and at sites that will ultimately become NCOs. This is consistent with our analysis of GFP::RMH-1 localization following induction of excessive DNA breaks by ionizing radiation (IR, 50Gy). We found that at 4 h post IR treatment, the number of RMH-1 foci in mid pachytene nuclei had risen significantly, suggesting that RMH-1 was loaded on many of the excess recombination intermediates produced by IR. To understand what would happen to those excess recombination intermediates positive for RMH-1, we analyzed gonads 8 h post IR treatment (after this time, the nuclei with the increased number of RMH-1 foci should have reached late pachytene). However, at this stage, the numbers were reduced to wild type numbers of foci in late pachytene nuclei (S7A–S7C Fig). Thus, we conclude that RMH-1 marks recombination intermediates that can be increased transiently by irradiation but will be repaired as NCOs, as the final number of marked CO sites is not affected.

### Crossover Promoting Function(s) of RMH-1

The robust accumulation of RMH-1 at CO sites, the dependence of its localization on pro-CO factors, and the presence of univalents in the *rmh-1* mutants together suggest that RMH-1 likely functions in promoting the CO outcome of initiated recombination events. Several lines of evidence provide further support for pro-CO role(s) for RMH-1. First, we tested whether *rmh-1* is required for normal localization of pro-CO factors by assessing the localization of GFP::COSA-1 in *rmh-1* mutants. This analysis revealed that only 75% of nuclei in *tn309* and 57% of nuclei in *jf54* had six COSA-1 foci (compared to 88% in wild-type) (Fig 4E and 4F), indicating a deficit of CO-designated sites in *rmh-1* mutants.

Furthermore, we observed several indications of a delay in pachytene progression in both *rmh-1* mutants. First, we assessed localization of ZHP-3, which normally localizes along the full length of the synaptonemal complex (SC) during mid pachytene, then retracts during late pachytene to a short stretch and, finally, to a single focus at the CO site in diplotene [66]; we found that ZHP-3 retraction was delayed in *rmh-1(jf54)* (S8A–S8D Fig). Second, we evaluated a readout of a checkpoint-like feedback mechanism that operates during *C*. *elegans* meiosis to coordinate meiotic progression with generation of CO-eligible recombination intermediates. A single chromosome pair that lacks a CO-eligible recombination intermediate can be detected and elicit a prophase progression delay, visible by the persistence of markers such as phospho-SUN-1 S8 on the nuclear envelope [67]. SUN-1 S8Pi staining was prolonged in *rmh-1* mutants, indicating a delay in prophase progression (S8E–S8H Fig), which is in line with delayed and/or impaired formation of CO-specific recombination intermediates. These data in combination with the observed reduction in COSA-1-marked CO-designated sites together indicate a role for RMH-1 in ensuring efficient CO designation.

Although the number of CO-designated sites is reduced in *rmh-1* mutants, CO designation still occurs to a large extent, as 57% to 75% of nuclei contain six COSA-1 foci. Moreover, the reduction in number of COSA-1-marked CO sites is not sufficient to account for the number of univalents observed. To calculate the expected number of DAPI bodies in diakinesis nuclei based on our quantification of COSA-1 foci in *rmh-1* mutants, we assumed that pachytene nuclei containing six COSA-1 foci would lead to diakinesis nuclei with six bivalents, pachytene nuclei containing five COSA-1 foci would lead to diakinesis nuclei with seven DAPI bodies (five bivalents and two univalents), and so on. This approach yields an expectation of 6.7 DAPI bodies at diakinesis for *rmh-1(jf54)* and 6.6 for *rmh-1(tn309)*, values that are significantly lower than those observed in the -1 oocytes of these mutants (Fig 1B and 1F). Thus, the number of univalents present at the end of meiotic prophase is higher than would be expected if all COSA-1-marked sites had successfully matured into COs.

To understand this excess of univalents, we investigated the organization of chromosomes in diakinesis. In wild type, the presence of a CO precursor triggers domain restructuring of the ensuing bivalent such that the axial proteins HTP-1/2 and LAB-1 become concentrated along the long arm of the bivalent, and the synapsis protein SYP-1 and the Aurora B kinase (AIR-2) are found on the short arm [68–70]. In *spo-11* or *msh-5* mutants, short and long arm markers overlapped on the univalents. In *rmh-1* mutants, however, we observed long and short arm markers localized to reciprocal domains on univalents (Fig 4G), suggesting that a CO site had been designated but failed to mature to actually link the homologs. Furthermore, quantification of DAPI bodies during progression through the diakinesis stage (-3, -2, and -1 oocyte) showed that they increased significantly (Fig 4H–4J). We observed that in the -3 oocyte, the number of DAPI bodies in *rmh-1* mutants (6.9 ± 0.7 DAPI bodies for *jf54* and 6.8 ± 0.9 for *tn309*) was significantly increased compared to wild type. Interestingly, the observed number of DAPI bodies in -3 oocytes of the mutants corresponds to the calculated number of DAPI bodies based on COSA-1 foci analysis. However, in both mutants, the number of DAPI bodies strongly increased during oocyte maturation. In *rmh-1(jf54)*, we observed 9.3 ± 1.4 DAPI bodies in the -1 oocytes, while the effect was milder but still significant in *rmh-1(tn309)* (7.4 ± 1.1 in -1 oocytes). We conclude that, in *rmh-1* mutants, connections between homologs may sometimes be lost after CO designation.

### Genetic Interactions with Mutations Affecting Structure-Specific Endonucleases

Dissociation of bivalents that appeared to have undergone CO designation suggested that RMH-1 might function to enforce CO bias in the resolution of recombination intermediates at CO sites. This hypothesis prompted us to examine double mutants carrying *rmh-1(jf54)* in combination with mutations in *xpf-1*, *mus-81*, or *slx-1*, which encode structure-specific endonucleases proposed to function in two separate and partially redundant meiotic resolution pathways [22–24], and *him-18* (*Slx4 ortholog)*, which encodes a nuclease scaffolding protein [71]. Dissociation of bivalents in *rmh-1(jf54)* was reduced by loss of *xpf-1*, *mus-81*, or *slx-*1 (Fig 4K), consistent with persistence of recombination intermediates; however, it is unclear whether such putative persistent intermediates are located at CO-designated sites and/or at other sites on the chromosomes. Furthermore, we observed an elevated incidence of unresolved bivalents linked by DNA bridges (Fig 4M and 4N) in these double mutants compared to the *xpf-1*, *mus-81*, or *slx-1* single mutants. Together with previous work showing that such linkages observed in nuclease-defective mutants are *spo-11*-dependent [22], these results suggest that loss of RMH-1 function may result in the accumulation of excess and/or aberrant recombination intermediates that require these structure-specific endonucleases for their resolution.

We also observed the occurrence of DNA bridges in the double *rmh-1(jf54); him-18* mutant, but, those linkages appeared with the same frequency as in the *him-18* single mutant. Furthermore, loss of *him-18* in *rmh-1(jf54)* did not appreciably suppress the dissociation of homologs (Fig 4K), in contrast to the outcomes with the single-nuclease mutants. We can reconcile these seemingly paradoxical observations by postulating (1) that unscaffolded nucleases may be able to act on recombination intermediates produced in *rmh-1* mutants to remove excess connections between homologs, and (2) that the presence of multiple unresolved intermediates connecting homologs (in *rmh-1; xpf-1*, *rmh-1 mus-81*, or *rmh-1 slx-1* mutants) might obscure detection of discrete bridges.

### Interdependent and Separable Functions of RMH-1 and HIM-6 (BLM Helicase)

As RMI1 was first identified as a component of a complex containing the RecQ helicase BLM and the topoisomerase TOP3, we used the yeast-two-hybrid assay to confirm interactions between RMH-1 and HIM-6 (the *C*. *elegans* BLM ortholog) and between RMH-1 and TOP-3 (S9A Fig). Furthermore, as several phenotypes of the *rmh-1* mutants resemble phenotypes seen in *him-6* mutants (S9B and S9C Fig) [46,53], we investigated the cytological and functional inter-relationships between RMH-1 and HIM-6.

**Fig 5:**
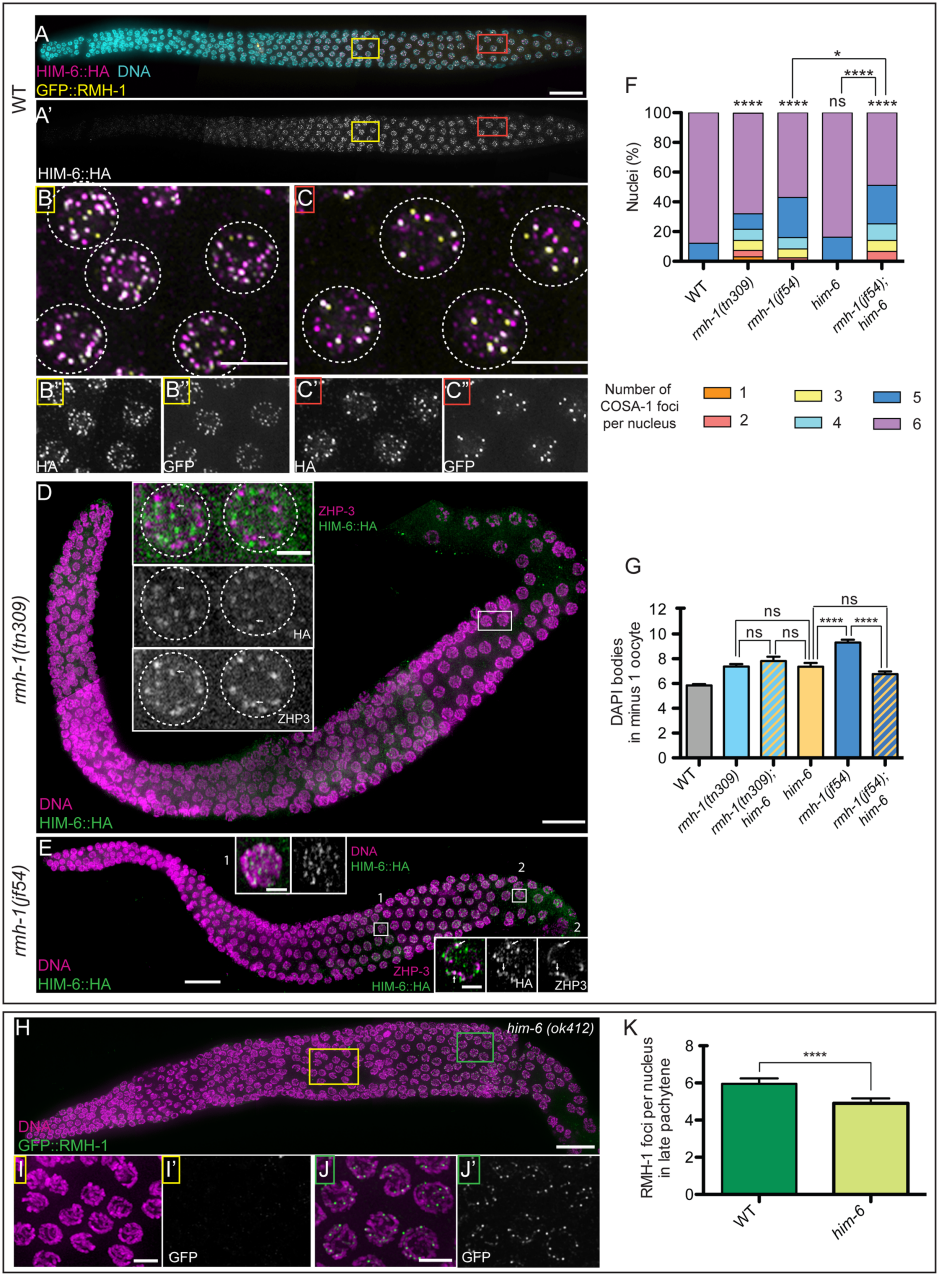
Separable and interdependent functions of RMH-1 and HIM-6 (BLM). (A-C) Comparison of RMH-1::GFP and HIM-6(BLM)::HA localization in wild-type germ cells. (B–B′′) In mid pachytene (MP) nuclei, a high number of GFP::RMH-1 and HIM-6::HA foci are present, and most colocalize. (C–C′′) In late pachytene (LP), HIM-6 and RMH-1 colocalize in bright foci, but additional HIM-6 signals are present at other sites. (D) In *rmh-1(tn309)*, HIM-6 foci are very small and faint (two gonads) or completely undetectable (three gonads). Inset: residual HIM-6 foci do not colocalize (white arrows) with ZHP-3. (E) In *rmh-1(jf54)*, HIM-6 is still present in foci. Insets for MP (1) and LP (2); in (2), HIM-6 foci can be found in proximity of ZHP-3 at some putative CO-designated sites; however, most do not colocalize. (F) Stacked bar graph showing the percentage of nuclei containing a defined number of COSA-1 foci either in WT (*n* = 428), *rmh-1(jf54)* (*n* = 291), *rmh-1(tn309)* (*n* = 329), *him-6(ok412)* (*n* = 204), or *rmh-1(jf54)*; *him-6* (*n* = 177). The data presented for WT, *rmh-1(jf54)*, and *rmh-1(tn309)* are the same as in Fig 4F. Difference in COSA-1 foci distribution was assessed by a Mann Whitney test: ns stands for not significant and **** *p* < 0.0001. (G) Quantification of the number of DAPI positive structures in the -1 oocyte for WT; single mutants (*rmh-1(tn309)- him-6(ok412)- rmh-1(jf54)) and* the double *rmh-1(tn309)*; *him-6* and *rmh-1(jf54)*; *him-6* (*n* = 15–36 nuclei per genotype). Data are represented as mean +/- SEM with ns (not significant) and **** *p* < 0.001. (H) In *him-6(ok412)*, RMH-1 is not detectable in MP (I,I′), but bright RMH-1 foci are present in LP (J,J′), albeit quantification (K) indicates that their numbers are reduced relative to WT (*p* < 0.001). Data are represented as mean +/- SD. Scale bar 20 μm for gonads, 5 μm for pachytene nuclei insets, and 2.5 μm for single nuclei.

To examine colocalization of RMH-1 and HIM-6, we used the CRISPR method to tag the *him-6* locus with a C-terminal human influenza hemagglutinin (HA) epitope. The tag did not compromise HIM-6 function, since neither an increase in embryonic inviability nor a Him phenotype was observed (S5 Fig). HIM-6 is found in few foci in early pachytene. The number of foci increases in mid pachytene and decreases again in late pachytene, reminiscent of the RMH-1 pattern. In mid pachytene, RMH-1 and HIM-6 substantially colocalized, and, in late pachytene, RMH-1 strictly colocalized with HIM-6; however, additional HIM-6 foci were also present (96.6 +/- 4.1% RMH-1 foci colocalize with HIM-6 in mid pachytene and 96.3 +/- 6.7% in late pachytene; Fig 5A–5C).

To investigate the interdependence of localization, we assayed localization of HIM-6::HA in *rmh-1* mutants. In *tn309*, HIM-6 was completely absent in most of the gonads we imaged. In the rest, HIM-6 was barely detectable, and when foci were seen, they were not at CO sites in late pachytene (Fig 5D). In *jf54*, HIM-6 foci were present in all gonads but seemed smaller and fainter in mid and late pachytene, suggesting that RMH-1 is required to stabilize and enrich HIM-6 in foci (Fig 5E). Although HIM-6 is sometimes found in proximity to CO site markers, these do not exhibit robust colocalization in the absence of *rmh-1* (Fig 5E).

Assessment of the localization of GFP::RMH-1 in the *him-6(ok412)* mutant revealed that *him-6* is required for RMH-1 localization in mid pachytene but not in late pachytene (Fig 5H–5J). This finding is consistent with the idea that RMH-1 may be present in genetically distinguishable complexes in mid and late pachytene stages. However, *him-6* is required for normal numbers of RMH-1 foci even in late pachytene (Fig 5K).

An important difference between *rmh-1* and *him-6* mutants is their impact on CO designation. A previous study showed that in the null allele of *him-6*, the abundance of COSA-1 foci was unaffected [46]; thus, the *him-6* null mutant appears to be proficient for CO designation. On the other hand, both *rmh-1* alleles impair CO designation, as evidenced by an elevated frequency of nuclei containing fewer than six COSA-1 foci (Fig 5F). Furthermore, the fact that CO designation is unaffected in a *him-6* null mutant but is impaired in an *rmh-1; him-6* double mutant implies that RMH-1 can assist in promoting CO designation in the absence of HIM-6.

A deficit of bivalent connections is a shared feature of *rmh-1* and *him-6* mutants. The phenotype is evident but less pronounced in *him-6* or *rmh-1(tn309)* (*p* < 0.05), while it is more prominent in *rmh-1(jf54)* (*p* < 0.01). The number of DAPI bodies in *rmh-1(tn309); him-6(ok412)* and the respective single mutants was not different. Interestingly, however, we found that the deficit of connections between homologs in *rmh-1(jf54); him-6(ok412)* oocytes was reduced compared to the *rmh-1(jf54)* single mutant (Fig 5G), despite the fact that COSA-1 foci were reduced in this double mutant. One possible explanation for this observation would be that the dissociation of bivalents observed in *rmh-1(jf54)* might be mediated by HIM-6. An alternative explanation is that the simultaneous loss of HIM-6 and RMH-1 may lead to persistence of unresolved recombination intermediates that maintain connections between the homologs.

### RMH-1 and HIM-6/ BLM Pachytene Foci Are Doublets/Elongated Structures

**Fig 6:**
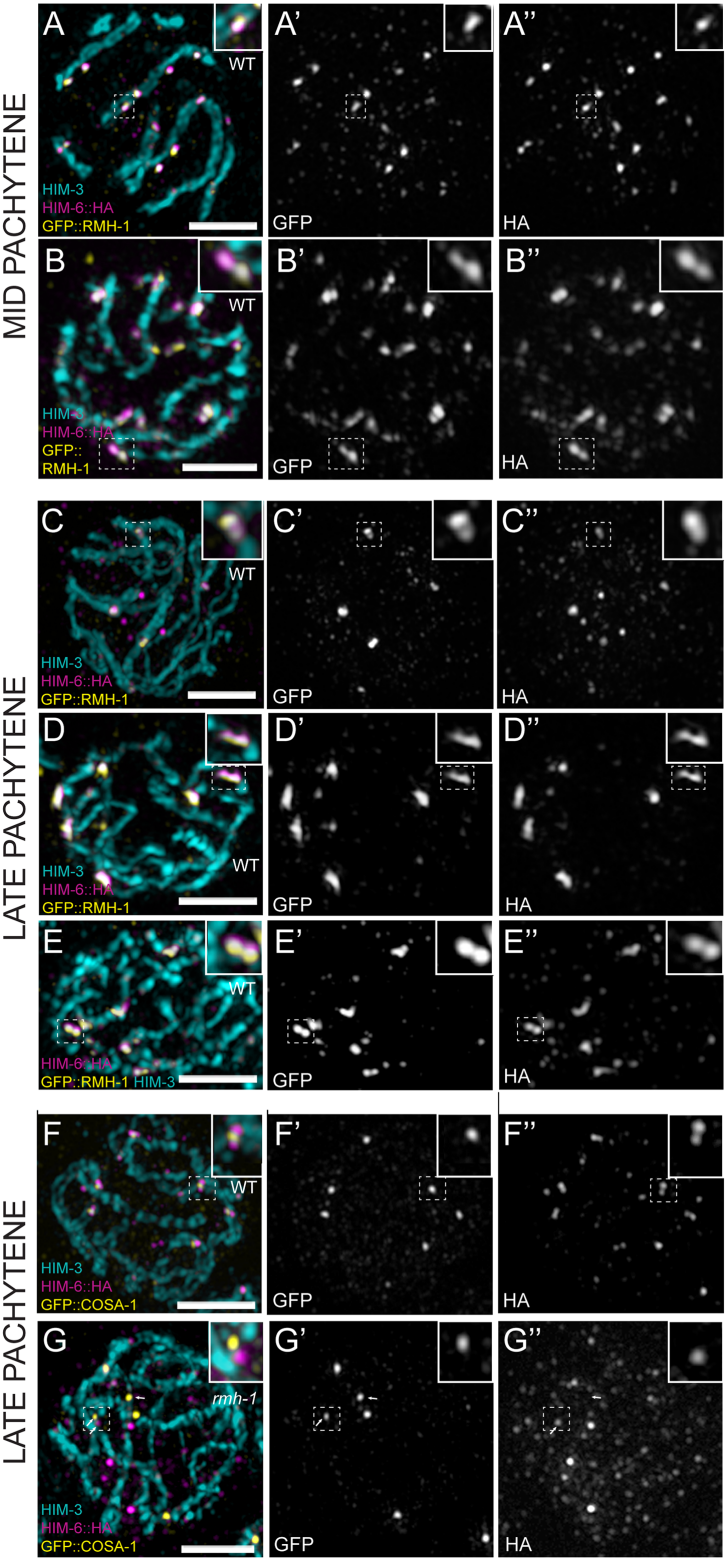
Foci of RMH-1 and BLM are resolved as doublets or elongated structures during the pachytene stage. (A–G) Single pachytene nuclei imaged by SIM. (A–B) In the WT, HIM-6 and RMH-1 colocalize in MP. Foci appear elongated or as a doublet (see insets). (C–E) In LP, RMH-1 and HIM-6 are concentrated at CO sites contained in a structure resolvable into a doublet (see insets). (F) Colocalization of HIM-6 and COSA-1 at CO sites in WT. (G) In *rmh-1(jf54)*, HIM-6 does not colocalize with COSA-1 at CO sites (white arrow) but can be found in close proximity (white arrows). Scale bar 2μm.

We investigated the foci structure of RMH-1 and HIM-6 foci using structured illumination microscopy (SIM). Both in mid pachytene and late pachytene, RMH-1 and HIM-6 signals were resolved into more complex structures (Fig 6). In mid pachytene, we observed foci that clearly exhibit a doublet structure and foci that show an elongated shape (Fig 6A and 6B). In late pachytene, RMH-1 and HIM-6 appear even more clearly as doublets (Fig 6C–6E). In both pachytene stages, we hypothesize that RMH-1 and HIM-6 foci mark similar recombination intermediates. The doublet structure is likely easier to observe in late foci as RMH-1 protein accumulates during pachytene progression, leading to larger foci in late pachytene. We measured the average distance between the foci peak in the three-dimensional stacks as an average of 227 +/- 46 nm. Interestingly, in *Drosophila*, the size of a recombination nodule has been estimated to around 100 nm [72]. It has also been shown that 1 kb of B-form DNA is 340 nm in length. We conclude that the structure we describe here is on an appropriate scale for flanking dHJs. We also observed that the late pachytene HIM-6 doublet structures flank a COSA-1 focus in the wild type (Fig 6F). Interestingly, in the *rmh-1(jf54*) mutant, HIM-6 appears as single foci and not as elongated or doublet structures in late pachytene (Fig 6G). This suggests that *rmh-1* is required to concentrate HIM-6 at CO sites and also to organize a complex structure surrounding late recombination intermediates.

### SMC-5 and RMH-1 Act Synergistically to Eliminate Illegitimate Interhomolog Connections

The mid pachytene localization of RMH-1 (and HIM-6) to numerous foci in excess of the eventual number of COs, together with the well-known role for the RTR complex in discouraging COs in other systems (see Introduction), led us to investigate the possibility that RMH-1 also functions at NCO sites during *C*. *elegans* meiosis. We focused on the possibility that RMH-1 might function in parallel with the SMC-5/6 complex, based on several considerations. First, several studies in budding yeast showed that in the absence of *Smc5* or *Smc6* during meiosis, COs form but joint molecules accumulate, leading to chromosome segregation defects [57–59]. Moreover, it was shown that Smc6 was required for the resolution of the joint molecules accumulating in absence of Sgs1, suggesting collaboration of pathways mediated through those two proteins [59]. Second, a prior study showed that the *C*. *elegans* SMC-5/6 complex is important for meiotic DSB repair under conditions in which interhomolog CO formation is abrogated and/or in mutant situations when intersister repair is the only option [73]. Moreover, the data of Bickel et al. are fully consistent with the SMC-5/6 complex participating in interhomolog NCO repair as well.

**Fig 7:**
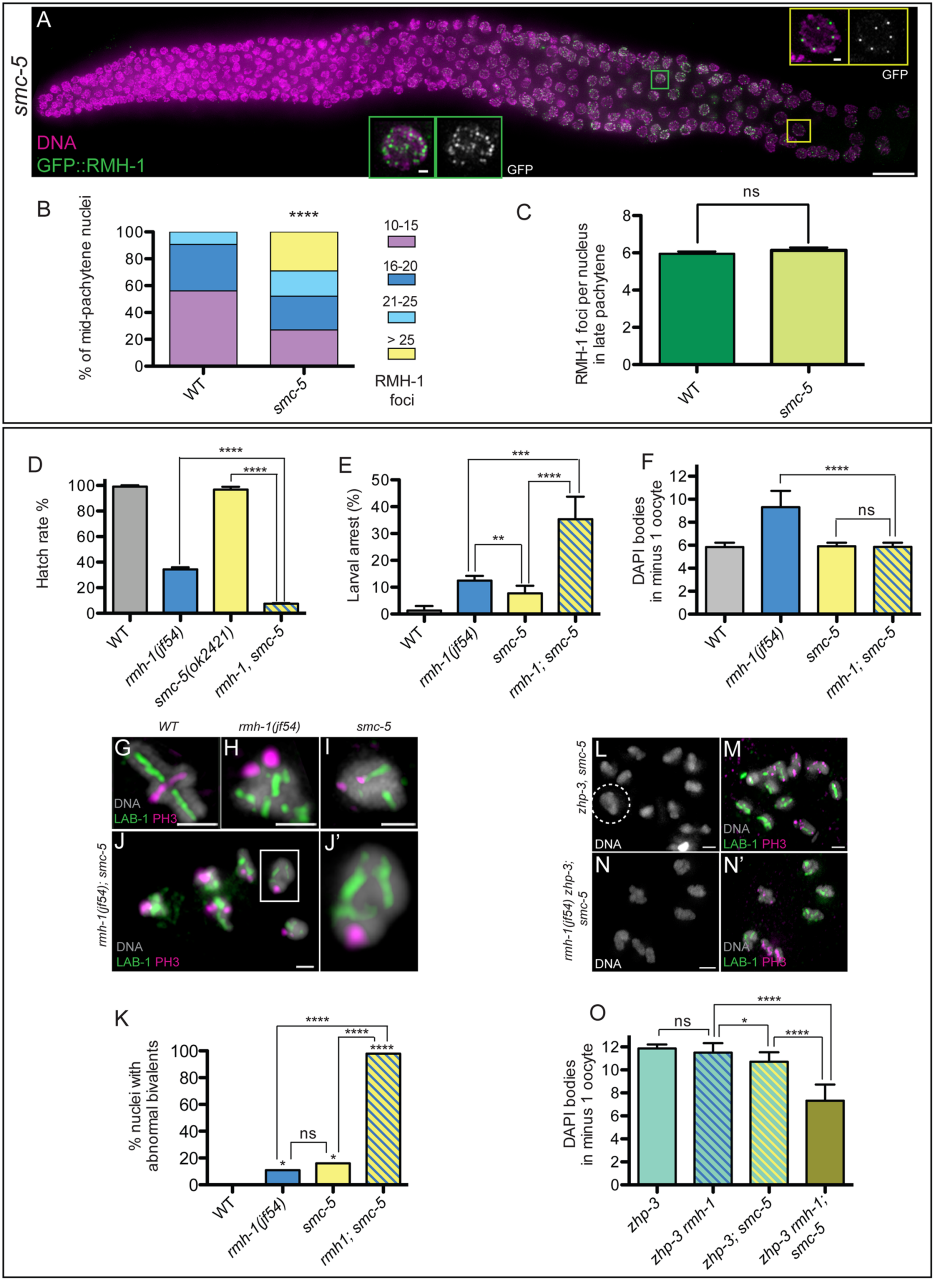
RMH-1 and SMC-5 cooperate to prevent accumulation of aberrant interhomolog connections. (A) In *smc-5(ok2421)*, RMH-1 foci increase in mid pachytene nuclei (MP) (green square), while in late pachytene (LP) (yellow square), a zone with fewer foci is still present, as in WT. (B) Quantification of RMH-1 foci in MP nuclei in WT (*n* = 205) and *smc-5(ok2421)* (*n* = 203). In the mutant, we frequently observe nuclei with more than 25 foci, never seen in the WT. Distribution of mid pachytene GFP::RMH-1 foci is significantly different between WT and *smc-5* mutant (Mann Whitney test, **** *p* < 0.0001). (C) Quantification of RMH-1 foci in LP nuclei; data are represented as mean +/- SD with ns (not significant). Quantification of hatch rate (D), larval arrest (E), and DAPI bodies in diakinesis oocytes (F) for WT, *rmh-1(jf54)*, *smc-5(ok2421)*, and *rmh-1(jf54); smc-5* (*n* = 35–45 hermaphrodites per genotype). Data are represented as mean +/- SD with ns (not significant) and * *p* < 0.05, ** *p* < 0.01, *** *p* < 0.001, **** *p* < 0.0001. (G–J) Images of individual diakinesis bivalents stained for long arm and short arm markers. Both *rmh-1(jf54)* and *smc-5* single mutants exhibit abnormally structured bivalents at low frequency (H,I). In *rmh-1; smc-5*, all diakinesis nuclei contain bivalents with abnormal structures; typical of these abnormalities is a side-by-side organization of the long arms of the bivalents (J′), presumably reflecting the presence of persistent interhomolog associations at NCO sites. (K) Quantification of the frequencies of diakinesis nuclei (-2 and -1) containing at least one abnormal bivalent (*n* = 13–25 nuclei per genotype). Data are represented as percentage with ns (not significant) and ** *p* < 0.01, *** *p* < 0.001, and **** *p* < 0.0001 (Chi^2^ test) (L–N) Images of chromosomes in diakinesis nuclei from *zhp-3; smc-5* and *rmh-1(jf54) zhp-3; smc-5* worms. Despite the absence of the canonical meiotic CO machinery component ZHP-3, fewer than 12 DAPI structures are observed in some *zhp-3; smc-5*–1 oocytes, indicating the presence of ectopic connections (L,M). Such ectopic connections occur at high frequency in the *rmh-1(jf54) zhp-3; smc-5* triple mutant (N–N′). The quantification is presented in (O) with *n* = 13–36 oocytes per genotype. Data are represented as mean +/- SD with ns (not significant), * *p* < 0.05 and **** *p* < 0.0001.

Several lines of evidence support the hypothesis that RMH-1 functions in parallel with the SMC-5/6 complex to antagonize accumulation of aberrant interhomolog connections. First, we observed a significant increase in RMH-1 foci in mid pachytene nuclei in the *smc-5* mutant; 29.1% of nuclei contained more than 25 foci, never seen in wild type (Fig 7A and 7B). However, late pachytene nuclei in the *smc-5* mutant had an average of six foci per nucleus, as in wild type (Fig 7C), suggesting that an excess of RMH-1-associated recombination intermediates accumulate in the *smc-5* mutant, but they are ultimately taken care of in agreement with the wild type viability of the single mutant (Fig 7D). Second, the *rmh-1(jf54); smc-5(ok2421)* double mutant showed both a synthetic decrease in embryonic hatch rates and increase in larval arrest (Fig 7D and 7E), indicating a strong genetic interaction between *rmh-1* and *smc-5* in the soma but also likely during meiosis (a similar genetic interaction is reported between *him-6* and *smc-5* in [74]). Third, quantification of the number of DAPI bodies in diakinesis oocytes in the *rmh-1; smc-5* double mutant revealed an average of 5.9 ± 0.3 DAPI bodies in the -1 oocyte (Fig 7F), consistent with the presence of connections between the homologs. However, cytological examination of chromosome organization revealed that these interhomolog connections were aberrant. Consistent with the low viability of this double mutant, bivalent organization was dramatically defective: both long arms (indicated by LAB-1 staining) were always found together, precluding the wild-type cross-shaped appearance of the bivalent (Fig 7G–7J). Such abnormal bivalents were already observed at a low frequency in each of the single mutants, but are now present in each diakinesis nucleus in the double mutant (Fig 7K). This result strongly suggests that *rmh-1* and *smc-5* cooperate to eliminate and/or prevent accumulation of connections between homologs at NCO sites. Finally, we found that simultaneous loss of both RMH-1 and SMC-5 results in a high frequency of interhomolog connections even in the absence of the conserved meiotic CO factor ZHP-3. Whereas COs and chiasmata are eliminated in the *zhp-3* single mutant [16], a small but significant reduction in DAPI bodies in -1 oocytes was observed in the *zhp-3; smc-5* mutant (Fig 7L, 7M and 7O). This ability to create aberrant homolog connections in a *zhp-3* mutant is consistent with the finding that a mutation affecting the budding yeast Smc5/6 complex has the ability to create connections between homologous chromosomes in a mutant lacking Zip3 (the ZHP-3 ortholog) [59]. Moreover, ectopic ZHP-3-independent connections occurred at very high frequency in a *zhp-3; rmh-1; smc-5* triple mutant, in which we observed an average of 7.3 DAPI bodies at diakinesis (Fig 7N and 7O). Together, our data indicate that *smc-5* and *rmh-1* act in parallel to antagonize MutS gamma-independent inter-homolog connections, supporting the conclusion that RMH-1 also functions at NCO sites.

### RMH-1 Discourages COs in the Center of Chromosomes

To assess the role of RMH-1 at NCO sites in more detail, we analyzed the localization of COs along the chromosomes. We used deep sequencing to compare CO frequency and CO distribution between the wild type and *rmh-1* mutants (*jf54* and *tn309*). We took advantage of the *C*. *elegans* Hawaiian (Hw) strain, which contains a high frequency of SNPs compared to the Bristol strain (wild type). Thus, we generated strains containing *rmh-1* mutants in combination with a subset of introgressed “Hawaii chromosomes”: chromosomes II and V for *rmh-1(jf54)* and chromosomes X, IV, and V for *rmh-1(tn309)*. (Introgression of all Hawaii chromosomes into the *rmh-1* mutants led to sterility).

**Fig 8:**
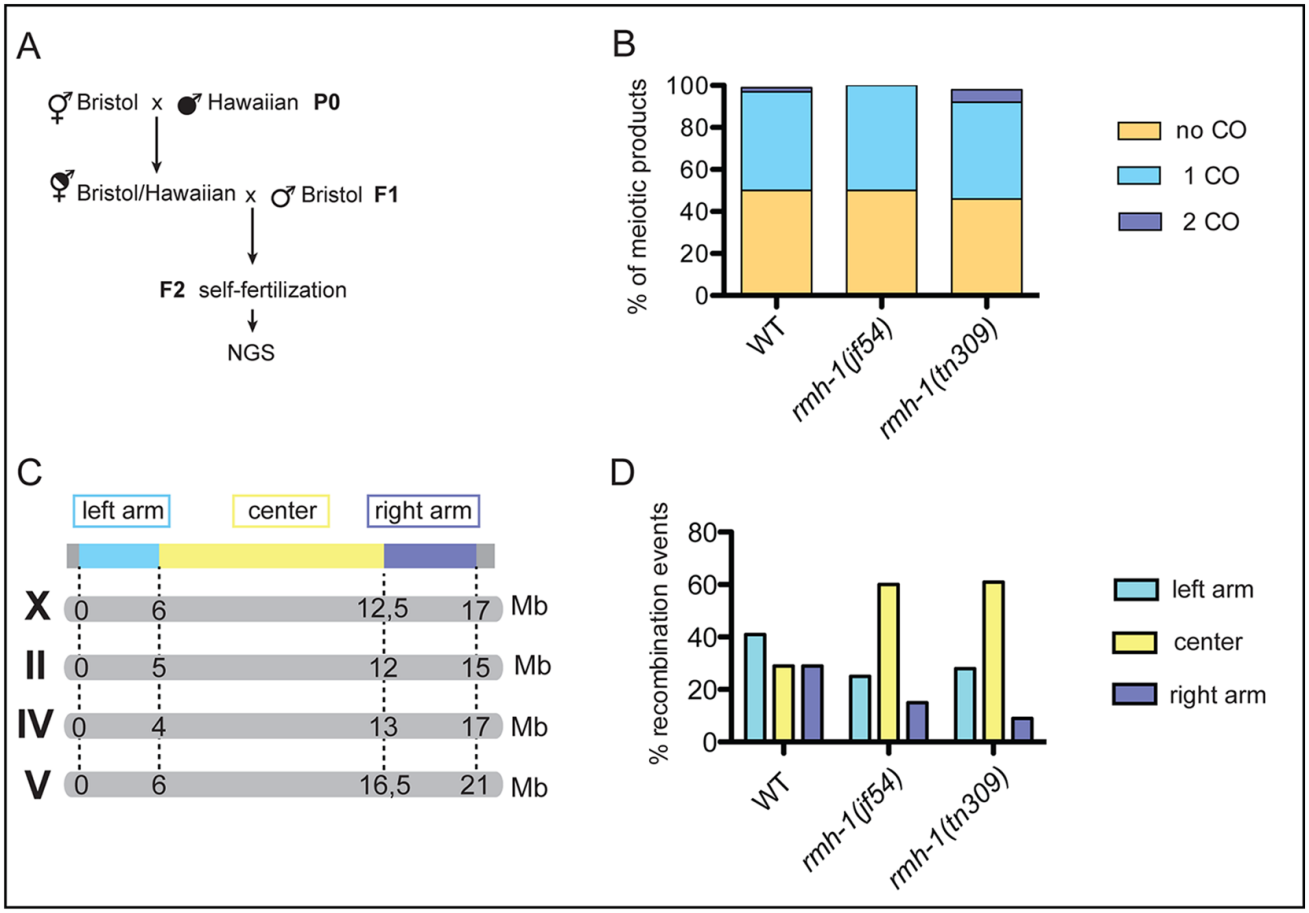
RMH-1 promotes the bias for CO formation on chromosome arms. (A) Schematics of crosses to obtain the progeny of singled F2 individuals subjected to Next Generation Sequencing (NGS) for SNP analysis. White insert indicates the WT (Bristol) background, and black insert indicates the Hawaiian background. (B) Quantification of the overall recombination frequencies for assayed chromosomes; stacked bar graph indicates the fraction of meiotic products with zero, one, or two COs. For WT (*n* = 36 chromatids), for *rmh-1(jf54)* (*n* = 40 chromatids), and for *rmh-1(tn309)* (*n* = 45 chromatids). The frequency of COs was not found to be different between WT and both mutants (Chi^2^ test). (C) Scheme of the different chromosomes used during the recombination assay. The chromosome domains (left arm in blue, center in yellow, and right arm in purple) are correlated with the physical map of each chromosome. (D) Locations of the recombination events (assayed for chromosomes X, IV, and V) in WT (*n* = 17 COs: three events on X, four on II, four on IV, and six on V), for *rmh-1(jf54)* (*n* = 20 COs: 11 events on II and 9 on V), and *rmh-1(tn309)* (*n* = 21 COs: nine events on X, nine on IV, and three on V); also see the Experimental Procedures section. The relative distribution of COs in the center versus arm domains differed from the WT for *tn309* (*p* = 0.046, Chi^2^ test) and for *jf54*, (p = 0.062, Chi^2^ test).

We sequenced all the parental strains (Bristol, Hw, and the “Hw introgressed *rmh-1* mutants”) and used them as references. Recombinants between the Bristol strain and Hw were generated as described in Fig 8A. We singled F2 animals and allowed them to self-fertilize for three to four generations and deep-sequenced DNA isolated from their progeny. Paired-end reads were mapped as described in the Experimental Procedures section. Bioinformatic analysis identified 166,928 homozygous unique SNPs in the Hw genome. To assess the chromosome composition in each recombinant, we used the homozygous unique SNPs of the Hw strain. We used SNPs located at least 400 bp apart to avoid having two or more SNPs per paired read. In the absence of recombination, two genotypes are expected in the offspring: fully homozygous (i.e., both homologous chromosomes come from the same parental strain) and fully heterozygous (each homolog comes from one parental strain). If recombination occurred, blocks of homozygosity and blocks of heterozygosity were detected. We identified the recombination break-points by analyzing the changes in heterozygosity along the chromosome (for more details, see the Experimental Procedures section).

We did not detect a significant difference between the mutants and wild-type control in the total frequencies of crossovers (Fig 8B). This observation contrasts with the reduction in COSA-1 foci observed in both mutants, and could potentially reflect sample size or the fact that viable progeny were used in the recombination assay; alternatively, it may reflect crossovers occurring at sites not marked by COSA-1 foci in the mutants. However, we found that the positions of the COs were shifted significantly toward the center of chromosomes in both mutants, in contrast to the preference observed in the wild type, in which the majority of COs occurred in the gene-sparse “arm” regions of the chromosomes (Fig 8C and 8D).

We conclude that *rmh-1* is required to discourage COs in the center of chromosomes. A shift of COs toward chromosome centers in the *rmh-1(jf54)* mutant was confirmed by using a PCR-based assay to genotype four SNPs along chromosome IV (S10 Fig).

A parallel study analyzed the recombination rate and CO position in the *him-6* mutant. Here, the entire brood was used (dead and living progeny) to determine recombination; similarly, a shift toward the chromosome centers was observed [74].

## Discussion

### Genetically Separable Roles for RMH-1 at CO and NCO Sites

**Fig 9:**
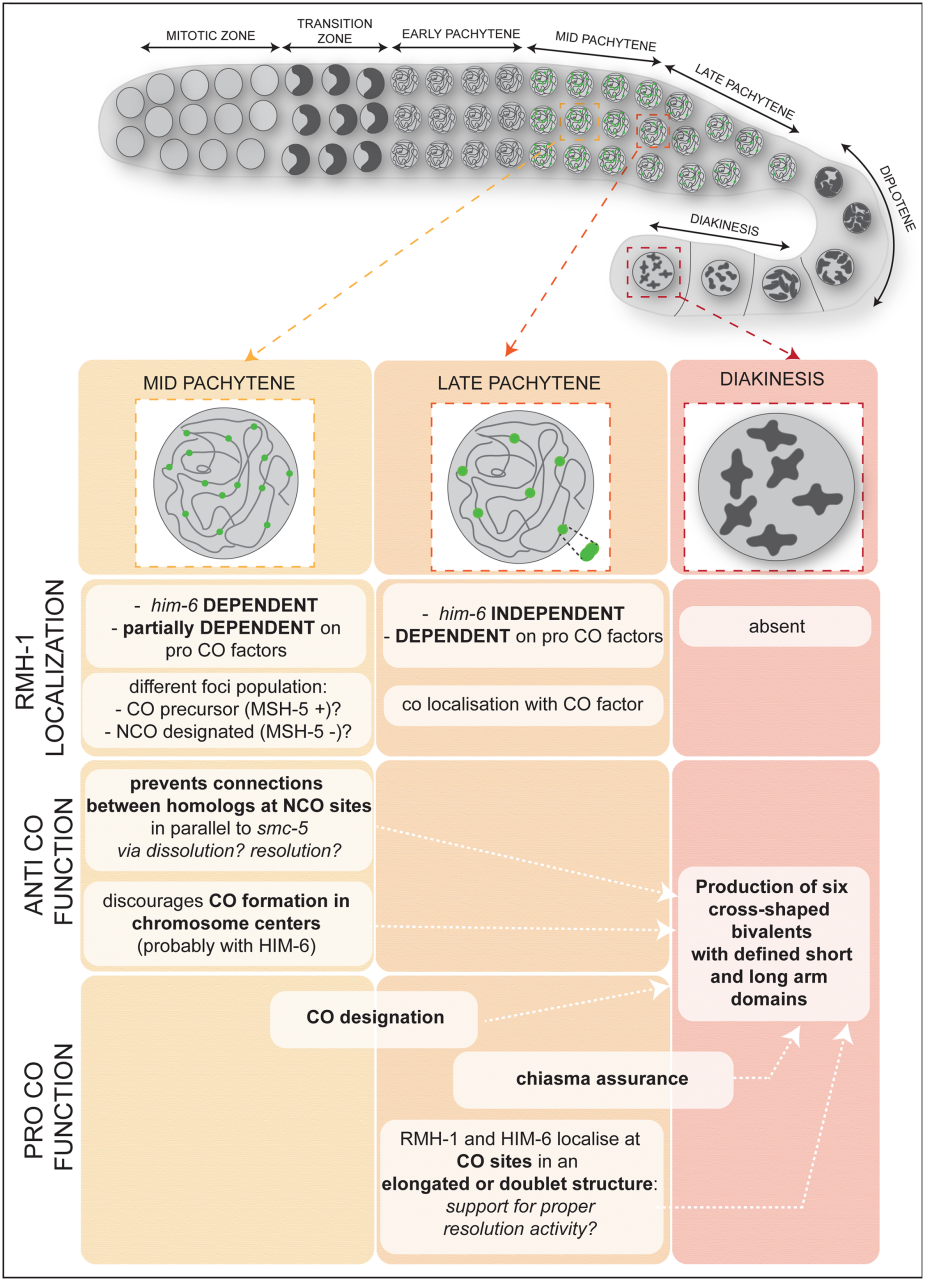
RMH-1 contributes to successful bivalent formation at several levels during prophase I. Top: scheme of *C*. *elegans* gonad showing prophase I divided in zones: transition zone (leptotene/zygotene), pachytene (early, mid, and late), diplotene, and diakinesis. Bottom: summary of localization and functions of RMH-1. Bold: key words referring to localization or function; italics: used when mechanistic insight is proposed. First row: localization of RMH-1: numerous foci in mid pachytene, six bright foci in late pachytene: RMH-1 is absent in diakinesis. Second row: genetic requirements and characteristics of RMH-1 localization. Third row: anti CO function: RMH-1 prevents accumulation of joint molecules and discourages CO formation in chromosome centers. Last row: pro CO functions: role of RMH-1 in CO designation, in assurance of chiasma formation and, finally, its potential function in supporting the geometry of recombination intermediates. We propose timing for the different functions based on our data and previous publications. However, the *C*. *elegans* gonad is a continuous production line of meiocytes, and we do not intend to imply sharper transitions between meiotic stages than exist.

Although protein complexes involving RMI1 and/or BLM helicase were initially recognized based on anti-CO activities, recent work in several systems has led to a growing realization that BLM and RMI1 play multiple roles during meiotic recombination, functioning both in antagonizing and promoting COs (for review, see [75] and Introduction). One current view for *Saccharomyces cerevisiae* is that the primary role of the RTR complex is as a “recombination intermediate chaperone” [41,44,45,55,56]. Under this model, RTR plays an indirect role in promoting COs during yeast meiosis, either by generating recombination intermediates recognized and processed by the Exo1-MutLγ pathway or by disassembling aberrant recombination intermediates, thereby helping to channel early recombination intermediates into either a MutSγ-dependent CO pathway or into the SDSA pathway for generating NCOs. Yeast Sgs1 has also been implicated in promoting resolution of CO intermediates under circumstances in which JM resolution is impaired [55]. A more prominent pro-CO role has been uncovered for HIM-6/BLM during *C*. *elegans* meiosis, in which loss of BLM function results in reduced COs [53,54]. It was proposed that HIM-6 functions to enforce a CO-biased outcome of resolution of CO intermediates; however, it was not clear whether this proposed pro-CO role for HIM-6 was direct or indirect [22,46]. Our combined genetic and cytological analysis provides a strong case that RMH-1 functions directly at both CO and NCO sites. For a summary, see Fig 9.

First, RMH-1 and HIM-6 colocalize at CO designated sites (Figs 3G–3K, 5A–5C and 6F), and this late pachytene localization is dependent on conserved pro-CO factors (Fig 4A–4D). Conversely, pro-CO factors can concentrate at presumptive CO sites in the absence of HIM-6 or RMH-1 (albeit less reliably in *rmh-1* mutants) consistent with RMH-1 and HIM-6 being recruited to CO designated sites to perform a late function in CO formation (Figs 4E, 4F and 5F). Moreover, SIM imaging of CO sites in late pachytene nuclei revealed that RMH-1 and HIM-6 colocalize in an elongated structure or closely juxtaposed doublets, potentially reflecting two separate populations of RTR complexes flanking the two junctions of a dHJ CO intermediate (Fig 6). The “doublet” organization leads us to suggest that RMH-1 and HIM-6 localized at CO sites may support the CO outcome of recombination intermediate resolution either by promoting an optimal geometry for endonucleases (resolvases) to position DNA cleavage, or by protecting the structure from unscheduled action of endonucleases. Our analysis with the truncation allele *tn309* raises the possibility that HIM-6 itself may contribute to inappropriate resolution when RMH-1 is absent.

Interestingly, RMH-1 and HIM-6 differ regarding the requirements for their recruitment at CO sites. Whereas RMH-1 is successfully recruited to most presumptive CO sites in *him-6* mutants (Fig 5H–5K), HIM-6 does not stably concentrate at the sites of pro-CO factors in *rmh-1* mutants (Fig 6G), raising the possibility that RMH-1 (or TOP-3 associated with RMH-1) may interact more directly with pro-CO factors at such sites. The absence of HIM-6 at CO sites in *rmh-1* mutants suggests that RMH-1 might provide a structural support to ensure the final processing of a mature recombination intermediate by limiting branch migration or the collapse of a putative dHJ and, thereby, influencing the outcome of resolution.

We provide multiple lines of evidence that RMH-1 also localizes to NCO sites and that its function(s) in NCO repair events are distinct from its pro-CO role(s). First, localization of RMH-1 during early and mid pachytene has different genetic requirements than its late pachytene localization at CO-associated foci: Early/mid pachytene RMH-1 foci can still form (albeit at reduced levels) in mutants defective for pro-CO factors, but are strictly dependent on HIM-6, suggesting that the intermediates present at early and late foci differ in structure (Figs 4A–4D and 5H–5K). Furthermore, we show that such foci can occur at elevated levels when numbers of DSB are increased or when alternative pathways for DSB repair are abrogated (Fig 7A and S7A–S7C Fig).

In mid pachytene, HIM-6 and RMH-1 proteins colocalize, but only a fraction colocalize with MSH-5 (Fig 3G–3I). We hypothesize that foci positive for all three proteins correspond to CO-eligible recombination sites, while those positive for only HIM-6 and RMH-1 may be processed as NCOs (perhaps by a decatenation reaction). The occurrence of chromosome bridges in *rmh-1; resolvase* double mutants could indicate either the accumulation of recombination intermediates due to defective dissolution or a more direct collaboration between RMH-1 and resolvases to produce COs and/or NCOs (Fig 4K–4M).

Together, our data suggest direct but distinct roles for RMH-1 in both CO and NCO repair during *C*. *elegans* meiosis.

### Functions of RMH-1 at CO and NCO Sites Collaborate to Prepare Chromosomes for Segregation During the Meiotic Divisions

Another important concept emphasized by our work analyzing the functions of RMH-1 during *C*. *elegans* meiosis is that a successful outcome of meiosis requires proper execution of the prescribed recombination events at both CO and NCO repair sites. Whereas COs serve as the basis of chiasmata that connect homologous chromosomes to enable them to orient toward opposite poles of the meiosis I spindle, efficient homolog segregation also depends on elimination of other recombination-based interactions between homologs that might impede their timely separation.

Beyond ensuring CO formation, RMH-1 has (at least) two roles in preparing homologous chromosome for segregation at meiosis I. First, RMH-1 functions in parallel with SMC-5 in eliminating excess associations between homologs (Fig 7). Importantly, we also demonstrate another major role for RMH-1 in ensuring that COs usually form in positions that favor reliable homolog segregation. In the absence of localized centromeres (due to the holocentric nature of *C*. *elegans* chromosomes), the position of the CO dictates multiple features of bivalent organization that are crucial for correct alignment and behavior of chromosomes [69]. This defines distinct long arm and short arm domains where cohesion will be retained or released at the meiosis I division and delineates how kinetochore components, motor proteins, and cell-division protein kinases associate with the chromosomes. We found that, whereas COs usually occur at off-center positions during wild-type meiosis, COs occurred more frequently near the centers of the chromosomes in *rmh-1* mutants (Fig 8 and S10 Fig). We speculate that an altered distribution of COs in an *rmh-1* mutant may reflect impaired execution at both CO and NCO repair sites. On the one hand, an impaired ability to impose CO-biased resolution at CO-designated sites results in intermediates at some of those sites being resolved as NCOs. On the other hand, impairment of a mechanism that would normally help dissociate intermediates to promote repair via SDSA may result in accumulation of DNA structures that require structure-specific endonucleases for their resolution, resulting in CO repair products at some sites that should have been NCOs. We have not observed a significant frequency of double COs in the mutants, suggesting that the misplaced COs are still subjected to interference. Interestingly, the SLX-1 resolvase is also required to suppress CO formation in the chromosome center [24]. It is appealing to speculate that RMH-1 might cooperate with SLX-1 in a locally defined region to promote NCO resolution.

We note that while CO positioning may be especially important in organisms like *C*. *elegans* that use COs to trigger differentiation of the bivalent, CO position is also very important during *Drosophila*, yeast, and human meiosis. in *Drosophila*, centromere proximal recombination events have been correlated to metaphase II nondisjunction [76]. In yeast, centromere proximal COs have been shown to trigger premature separation of sister chromatids (PSSC). Interestingly, *sgs1* mutants exhibit reduced spore viability, potentially due to an excess of COs near the centromere leading to PSSC [77]. In human meiosis, COs located very near to either telomeres or centromeres are associated with elevated risk of aneuploidies, e.g., [78–82].

## Experimental Procedures

Cultivation of worms followed standard protocols [83]. All experiments were performed at 20°C. N2 Bristol was used as wild type. Experiments using GFP::RMH-1 always included the wild-type *rmh-1* gene, if not indicated otherwise. Strains are listed in S1 Text. Raw data for the figures can be found in S1 Table.

### Cytological Preparations and Immunofluorescence Analysis

Dissection and immunostaining was performed as described in [67] with modifications when using SYP-1, LAB-1, and S10 phospho-histone 3: dissection and fixation was performed in 1x EBT instead of PBS 1X [66]. Adults 24 h post L4 were dissected. Images are projections of stacks encompassing whole nuclei. For details on antibodies and microscopy, refer to S1 Text.

See also S1 Text for *rmh-1* allele isolation and characterization, protein tagging (RMH-1, HIM-6::HA), generation of the *rmh-2* knockout, irradiation conditions, yeast two hybrid assays, photoconversion experiments, and recombination assays.

## Supporting Information

S1 Fig Identification of *rmh-1* (RMI homolog 1) mutants. (A) Alignment of the N- and C-terminal parts of RMI1 homologs: human (Hs), *C*. *elegans* (Ce), *Arabidopsis thaliana* (At), *Saccharomyces pombe* (Sp), and *Saccharomyces cerevisae* (Sc), with the DUF1767 domain (green frame) as described in [47] and the OB domains (orange frame) as described in [35]. (B) The mutation in *rmh-1(jf54)* affects the splice donor site of the first exon. Schematics of PCR primers used to analyze the structures of transcripts produced by the *rmh-1* locus. The spliced version corresponds to the wild type. In the unspliced version, intron 1 is present. Two different guanidines (three base pairs apart), present in exon 1, were used as cryptic splicing donor sites to substitute the original G mutated site in *rmh-1(jf54)*. Those versions led to deletions of intron 1 and a small portion of exon 1 (conserved amino acid in the DUF domain), as illustrated in (A). (C) Quantification of transcript abundance (four replicates). The percentage of transcripts represents the fraction of the total population of transcripts in one genotype (wild type or *jf54*). Data are represented as mean +/- SD (*** *p* < 0.001 and **** *p* < 0.0001). (D) Schematics of the protein products predicted for the different mutant alleles of *rmh-1*: *jf92*, *tn309*, and *jf54*.
(TIF)

S2 Fig Requirements for GFP::RMH-1 localization. (A and A′) DSBs are required for RMH-1 localization throughout pachytene. In *spo-11(me44)*, which lacks meiotic DSBs, GFP:: RMH-1 is absent from DNA, although a few late pachytene nuclei contain one focus of protein (see inset). (B and C) Gonad from an *rmh-1(jf92)* worm expressing a *gfp*::*rmh-1* transgene containing the *rmh-1(jf54)* point mutation (B and B′) or the *gfp*::*rmh-1(tn309)* point mutation (C and C′). In both cases, GFP::RMH-1 is not detected in foci in the gonad. The fact that the transgene *gfp*::*rmh-1(tn309)* recapitulates the phenotype of the *tn309* allele (in a deletion background) proves expression of the transgene. Scale bars 20 μm for full gonad and 5 μm for insets.
(TIF)

S3 Fig *rmh-1* mutants exhibit a mixture of bivalents and univalents in diakinesis. (A) Quantification of the average volume of a bivalent (assessed in the wild type) and a univalent (assessed in the *msh-5 (me23)* mutant in the -1 oocyte (*n* = 13 nuclei for both genotypes). Data are presented as mean +/- SD with *p* < 0.0001. (B) Classification of the DAPI structures found in *rmh-1(jf54)* and *rmh-1(tn309)*. Classes (fragment < univalent < bivalent < chromatin masses) were defined by the volume of the structure (*n* = 13 nuclei for wild type, *msh-5(me23)*, and *rmh-1(tn309)*, and *n* = 12 nuclei for *rmh-1(jf54)*).
(TIF)

S4 Fig *rmh-1* is not required for pairing or synapsis. (A) Staining for HIM-8 (X chromosome pairing center binding protein) and (B) FISH with the 5S ribosomal locus (chromosome V) to follow pairing in *rmh-1(jf54)*. (D) Quantification of nuclei with a paired signal of HIM-8 in six equal zones in the gonad, as shown in (C) (*n* = 52–96 nuclei per zone for each genotype). Distribution of paired HIM-8 signals is not different between the wild type and mutant (Chi^2^ test). (E and G) Staining for SYP-1 protein (transverse filament of the SC) on wild type (E) or *rmh-1(jf54)* gonads (G). (F and H) Staining for HTP-3 (axial element of the SC) on wild type or *rmh-1(jf54)* gonads (H). Scale bars 5 μm.
(TIF)

S5 Fig GFP::RMH-1 and HA::HIM-6 are functional transgenes. (A) Quantification of embryonic hatch rates and (B) the frequency of male offspring among the progeny of wild type, *rmh-1(jf92); gfp*::*rmh-1* (*n* = 35 hermaphrodites), *him-6*::*HA* (*n* = 45), and *gfp*::*rmh-1*; *him-6*::*HA* (*n* = 42). Data are represented as mean +/- SD (* for *p* < 0.05, **** for *p* < 0.0001, and ns stands for not significant).
(TIF)

S6 Fig Time course of recombination markers in prophase I. (A) Scheme of a *C*. *elegans* gonad showing the different meiotic stages: transition zone, pachytene (early, mid, and late), diplotene, and diakinesis. (B) Quantification of the average number of RAD-51, GFP::RMH-1, or GFP::COSA-1 foci per nucleus in the different meiotic zones of the gonad (*n* = 3 gonads). Data are represented as mean +/- SEM.
(TIF)

S7 Fig Mid and late pachytene RMH-1 foci are differently regulated. (A) Mid pachytene (MP) nuclei (white square) contain more RMH-1 foci than the wild type, 4 h after 50 Gy irradiation. (B) Stacked bar graph showing the percentage of MP nuclei containing a defined number of RMH-1 foci: unirradiated wild type (*n* = 205) or after irradiation (*n* = 228). Distribution of GFP::RMH-1 differed between irradiated and unirradiated (Mann-Whitney test, *p* < 0.0001). (C) Quantification of the average number of RMH-1 foci per nucleus in late pachytene in unirradiated wild type (*n* = 180) and 8 h post irradiation (*n* = 86). No difference is observed. Data are represented as mean +/- SD. (D and E) RMH-1 localization in *zhp-3(jf61)* or *msh-5(me23)* mutants. In both cases, RMH-1 foci are reduced and fainter in MP (insets) and absent in late pachytene (LP). Scale bars 20 μm for gonad and 5 μm for insets.
(TIF)

S8 Fig Prophase progression is delayed in *rmh-1*. (A-D) Staining for SYP-1 and ZHP-3 on wild type (A) and *rmh-1(jf54)* (B). In late pachytene in wild type, ZHP-3 marks the putative CO sites (A′ and A′′), while in *rmh-1(jf54)*, ZHP-3 is still present on the chromatin following the SC staining as stretches (D–D′′). The asymmetric retraction of SYP-1 is also delayed (C–C′′). (E–G) The SUN-1 S8 Pi positive region is extended in *rmh-1*. (H) Quantification of the length of the gonad zone positive for SUN-1 S8 Pi, expressed in percentage of length positive for the marker with regard to the length of zone from meiotic entry to diplotene (*n* = 3 gonads per genotype). Data are represented as mean +/- SD with (*** for *p* < 0.001 and **** for *p* < 0.0001). Scale bars 20 μm for gonad and 5 μm for insets.
(TIF)

S9 Fig Interplay between RMH-1 and HIM-6. (A) Yeast two-hybrid assays for protein interactions between RMH-1, HIM-6, and TOP-3. Full-length RMH-1 interacts with TOP-3 (row 1) and with HIM-6 (row 2), respectively. A truncation protein of RMH-1 containing only the N-terminal part with the DUF and OB1 domains (reminiscent of *tn309*) interacts with TOP-3 (row 5). Auto-activation of both bait and prey vectors were tested in lanes 6 to 10. Interactions were scored by growth on SC-Leu-Trp-His plates (left panel) and growth on SC-Leu-Trp plates (right panel). Quantification of the percentage of hatch rate (B) and males in offspring (C) for wild type (*n* = 45 hermaphrodites), single mutants (*rmh-1(tn309)* (*n* = 45)—*him-6(ok412)* (*n* = 35)—*rmh-1(jf54)* (*n* = 45)), and the double mutants *rmh-1(tn309); him-6* (*n* = 43) and *rmh-1(jf54); him-6* (*n* = 45). Data are represented as mean +/- SD with ns (not significant) and * *p* < 0.05, ** *p* < 0.01, *** *p* < 0.001, **** *p* < 0.0001.
(TIF)

S10 Fig COs are shifted toward the chromosome center in *rmh-1*. (A) Schematics of crosses to obtain the F2 individuals used for PCR-based SNP analysis. White insert indicates the wild type (Bristol) background, and black insert indicates the Hawaiian background. (B) Scheme of chromosome IV and location of the SNPs used during the recombination assay. SNP A is localized on the left arm, B is at the junction between left arm and the center, C is at the junction between the center and right arm, and D is on the right arm. (C) Recombination frequencies on chromosome IV left arm between SNP A and B for wild type (*n* = 94 individuals) and *rmh-1(jf54)* (*n* = 96 individuals). (D) Recombination frequencies on chromosome IV center between SNP B and C for wild type (*n* = 94 individuals) and *rmh-1(jf54)* (*n* = 96 individuals). COs were shifted to the centers of the chromosome in the mutant. (E) Recombination frequencies on chromosome IV right arm between SNPs C and D for wild type (*n* = 94 individuals) and *rmh-1(jf54)* (*n* = 96 individuals). (C–E) The column “theoretical” corresponds to the expected recombination frequency based on the published genetic distance (http://www.wormbase.org). Data are represented as percentage with ns for not significant, * for *p* < 0.05, and ** for *p* < 0.01 (Chi^2^ test on raw data). (F) Graph illustrating the percentage of CO events along chromosome IV for wild type and *rmh-1(jf54)*. In the wild type, COs are concentrated to chromosome arms, whereas in the mutant, they are more concentrated at the center.
(TIF)

S1 Table Raw data. (XLSX)

S1 Text Supporting information materials and methods. Detailed descriptions are provided for the strains used and generated in this study, antibodies and imaging methods, FISH procedure, identification of the *rmh-1* alleles, generation of *rmh-1*, *rmh-2* alleles and him6::HA strain by CRISPR method, the tagging of RMH-1, RT-PCR procedure, measurements of DAPI bodies in diakinesis, the Y2H assay, the recombination assays by PCR-based SNP mapping, and deep sequencing.
(DOCX)

## Abbreviations

BLM: Bloom syndrome protein
CO: crossover
dHJ: double Holliday junction
DSB: double strand break
DUF: domain of unknown function
HA: human influenza hemagglutinin
Him: high incidence of males
HR: homologous recombination
Hw: Hawaiian
IR: ionizing radiation
JM: joint molecules
NCO: non-crossover
NGS: Next Generation Sequencing
OB: oligonucleotide/oligosaccharide-binding
PSSC: premature separation of sister chromatids
RMH-1: RMI1 homolog-1
RTR: RecQ helicase–topoisomeraseIII–Rmi1
SC: synaptonemal complex
SDSA: synthesis-dependent strand annealing
SIM: structured illumination microscopy
RMH-2: RMI1 homolog 2
