# Supplementary figures and images for: Separable Roles for a Caenorhabditis elegans RMI1 Homolog in Promoting and Antagonizing Meiotic Crossovers Ensure Faithful Chromosome Inheritance
Source: PLoS Biol. 2016 Mar 24;14(3):e1002412. doi: 10.1371/journal.pbio.1002412 (PMC4807110; doi:10.1371/journal.pbio.1002412)

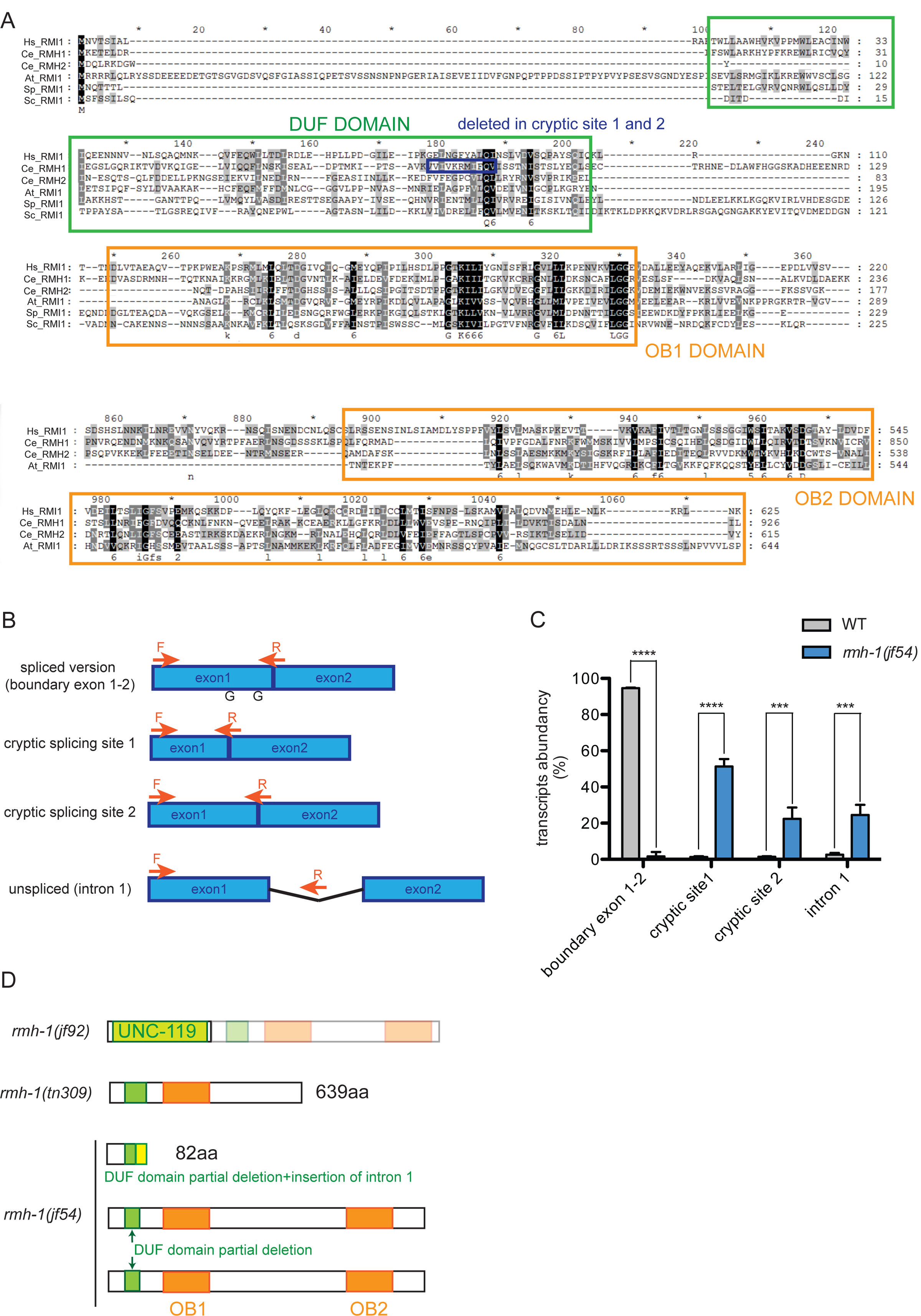

Supplement: S1 Fig — (A) Alignment of the N- and C-terminal parts of RMI1 homologs: human (Hs), C. elegans (Ce), Arabidopsis thaliana (At), Saccharomyces pombe (Sp), and Saccharomyces cerevisae (Sc), with the DUF1767 domain (green frame) as described in [47] and the OB domains (orange frame) as described in [35]. (B) The mutation in rmh-1(jf54) affects the splice donor site of the first exon. Schematics of PCR primers used to analyze the structures of transcripts produced by the rmh-1 locus. The spliced version corresponds to the wild type. In the unspliced version, intron 1 is present. Two different guanidines (three base pairs apart), present in exon 1, were used as cryptic splicing donor sites to substitute the original G mutated site in rmh-1(jf54). Those versions led to deletions of intron 1 and a small portion of exon 1 (conserved amino acid in the DUF domain), as illustrated in (A). (C) Quantification of transcript abundance (four replicates). The percentage of transcripts represents the fraction of the total population of transcripts in one genotype (wild type or jf54). Data are represented as mean +/- SD (*** p < 0.001 and **** p < 0.0001). (D) Schematics of the protein products predicted for the different mutant alleles of rmh-1: jf92, tn309, and jf54. (TIF) [file pbio.1002412.s001.tif]

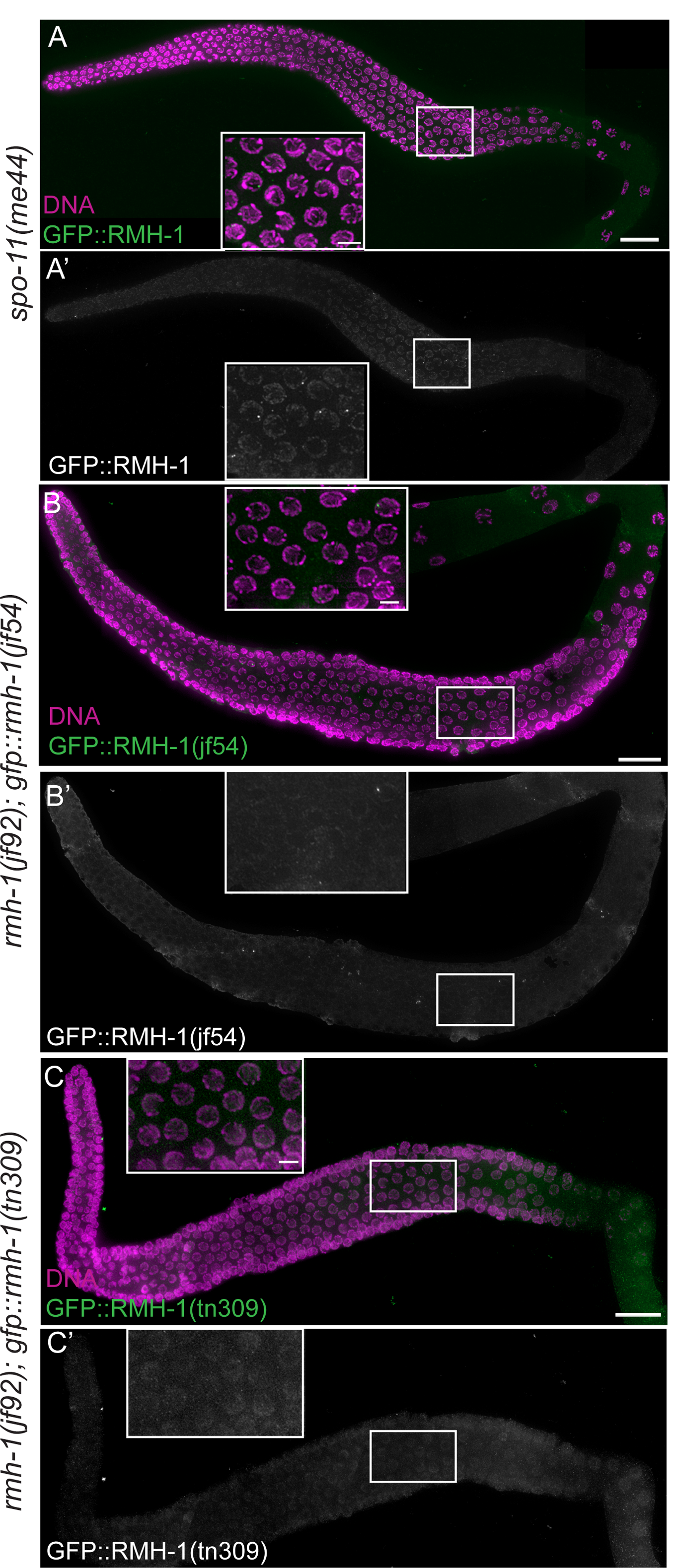

Supplement: S2 Fig — (A and A′) DSBs are required for RMH-1 localization throughout pachytene. In spo-11(me44), which lacks meiotic DSBs, GFP:: RMH-1 is absent from DNA, although a few late pachytene nuclei contain one focus of protein (see inset). (B and C) Gonad from an rmh-1(jf92) worm expressing a gfp::rmh-1 transgene containing the rmh-1(jf54) point mutation (B and B′) or the gfp::rmh-1(tn309) point mutation (C and C′). In both cases, GFP::RMH-1 is not detected in foci in the gonad. The fact that the transgene gfp::rmh-1(tn309) recapitulates the phenotype of the tn309 allele (in a deletion background) proves expression of the transgene. Scale bars 20 μm for full gonad and 5 μm for insets. (TIF) [file pbio.1002412.s002.tif]

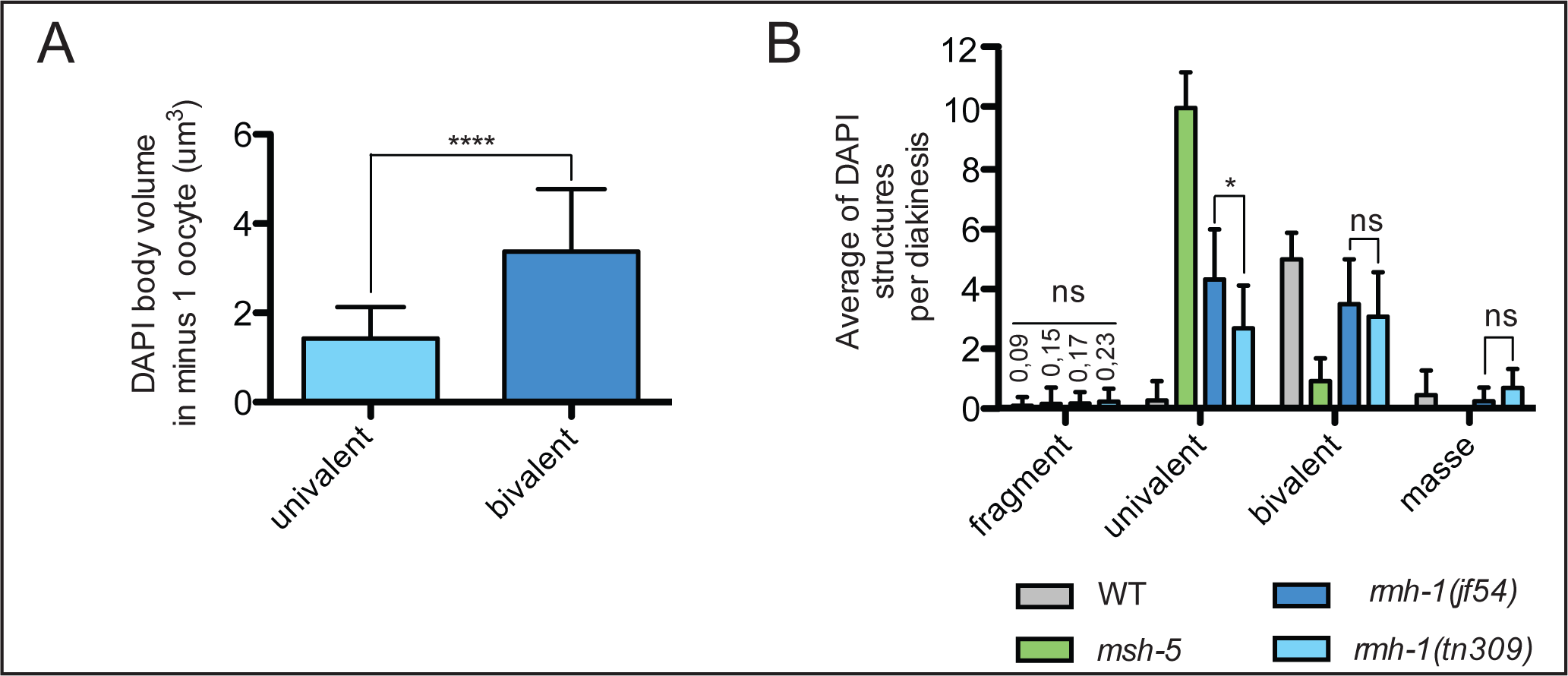

Supplement: S3 Fig — (A) Quantification of the average volume of a bivalent (assessed in the wild type) and a univalent (assessed in the msh-5 (me23) mutant in the -1 oocyte (n = 13 nuclei for both genotypes). Data are presented as mean +/- SD with p < 0.0001. (B) Classification of the DAPI structures found in rmh-1(jf54) and rmh-1(tn309). Classes (fragment < univalent < bivalent < chromatin masses) were defined by the volume of the structure (n = 13 nuclei for wild type, msh-5(me23), and rmh-1(tn309), and n = 12 nuclei for rmh-1(jf54)). (TIF) [file pbio.1002412.s003.tif]

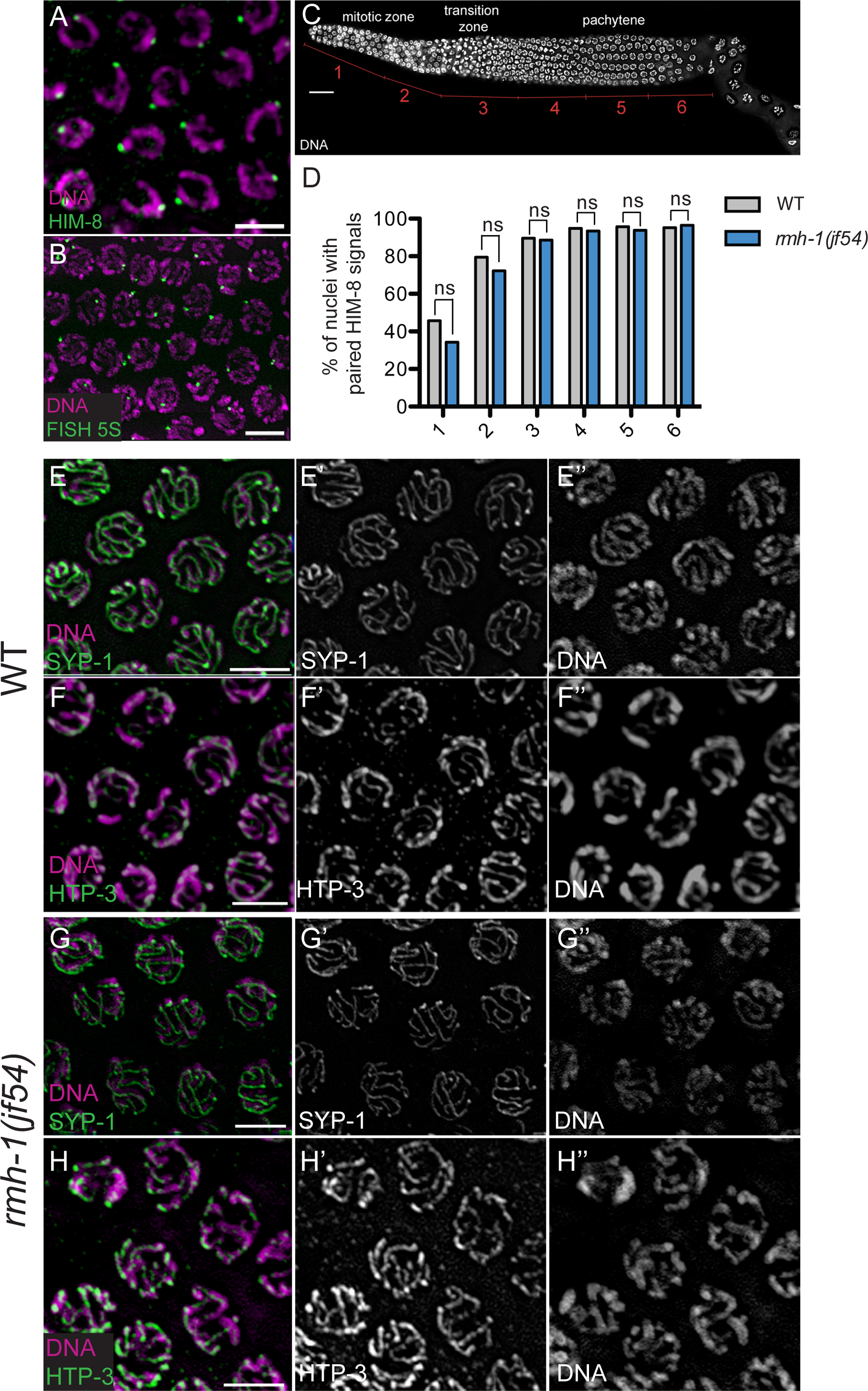

Supplement: S4 Fig — (A) Staining for HIM-8 (X chromosome pairing center binding protein) and (B) FISH with the 5S ribosomal locus (chromosome V) to follow pairing in rmh-1(jf54). (D) Quantification of nuclei with a paired signal of HIM-8 in six equal zones in the gonad, as shown in (C) (n = 52–96 nuclei per zone for each genotype). Distribution of paired HIM-8 signals is not different between the wild type and mutant (Chi2 test). (E and G) Staining for SYP-1 protein (transverse filament of the SC) on wild type (E) or rmh-1(jf54) gonads (G). (F and H) Staining for HTP-3 (axial element of the SC) on wild type or rmh-1(jf54) gonads (H). Scale bars 5 μm. (TIF) [file pbio.1002412.s004.tif]

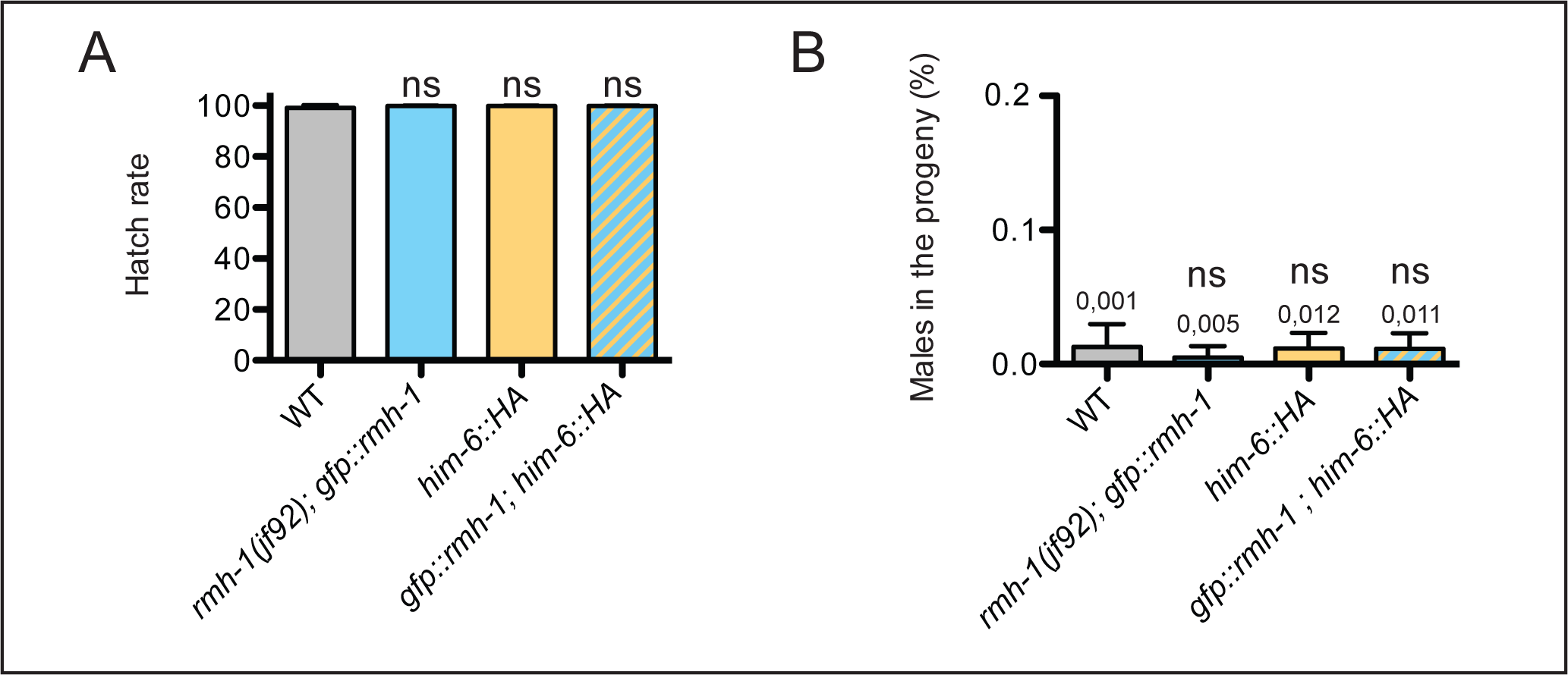

Supplement: S5 Fig — (A) Quantification of embryonic hatch rates and (B) the frequency of male offspring among the progeny of wild type, rmh-1(jf92); gfp::rmh-1 (n = 35 hermaphrodites), him-6::HA (n = 45), and gfp::rmh-1; him-6::HA (n = 42). Data are represented as mean +/- SD (* for p < 0.05, **** for p < 0.0001, and ns stands for not significant). (TIF) [file pbio.1002412.s005.tif]

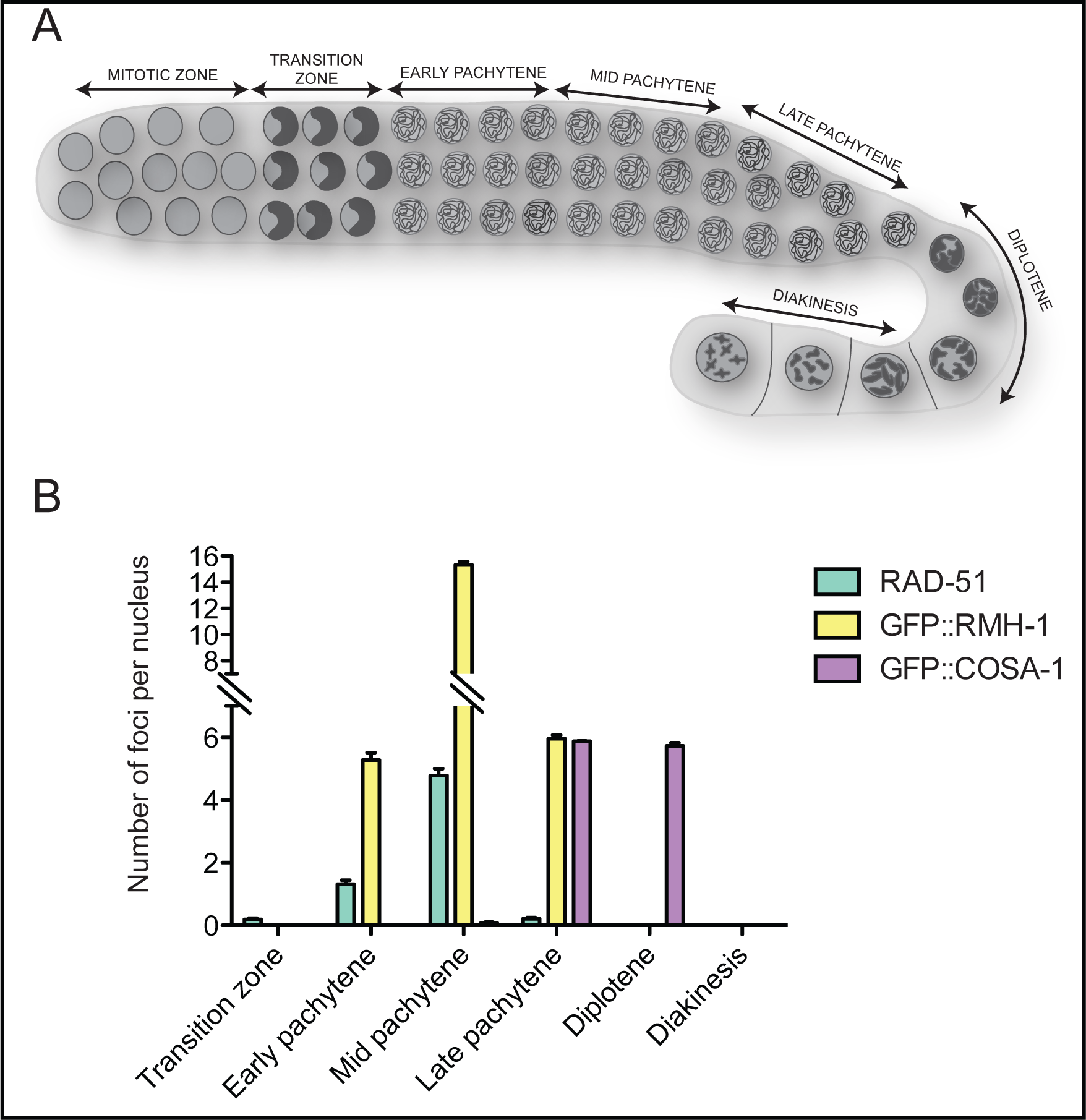

Supplement: S6 Fig — (A) Scheme of a C. elegans gonad showing the different meiotic stages: transition zone, pachytene (early, mid, and late), diplotene, and diakinesis. (B) Quantification of the average number of RAD-51, GFP::RMH-1, or GFP::COSA-1 foci per nucleus in the different meiotic zones of the gonad (n = 3 gonads). Data are represented as mean +/- SEM. (TIF) [file pbio.1002412.s006.tif]

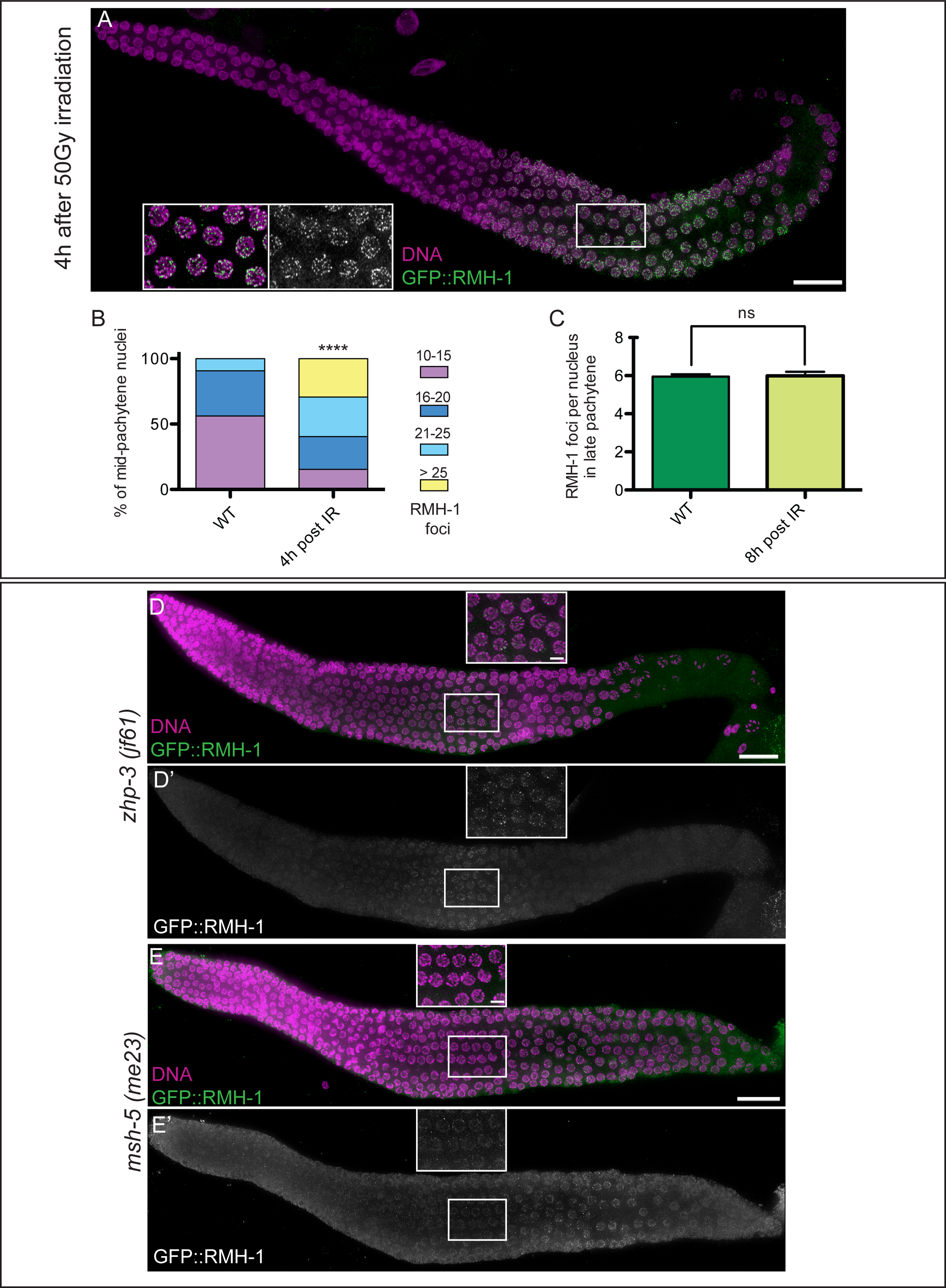

Supplement: S7 Fig — (A) Mid pachytene (MP) nuclei (white square) contain more RMH-1 foci than the wild type, 4 h after 50 Gy irradiation. (B) Stacked bar graph showing the percentage of MP nuclei containing a defined number of RMH-1 foci: unirradiated wild type (n = 205) or after irradiation (n = 228). Distribution of GFP::RMH-1 differed between irradiated and unirradiated (Mann-Whitney test, p < 0.0001). (C) Quantification of the average number of RMH-1 foci per nucleus in late pachytene in unirradiated wild type (n = 180) and 8 h post irradiation (n = 86). No difference is observed. Data are represented as mean +/- SD. (D and E) RMH-1 localization in zhp-3(jf61) or msh-5(me23) mutants. In both cases, RMH-1 foci are reduced and fainter in MP (insets) and absent in late pachytene (LP). Scale bars 20 μm for gonad and 5 μm for insets. (TIF) [file pbio.1002412.s007.tif]

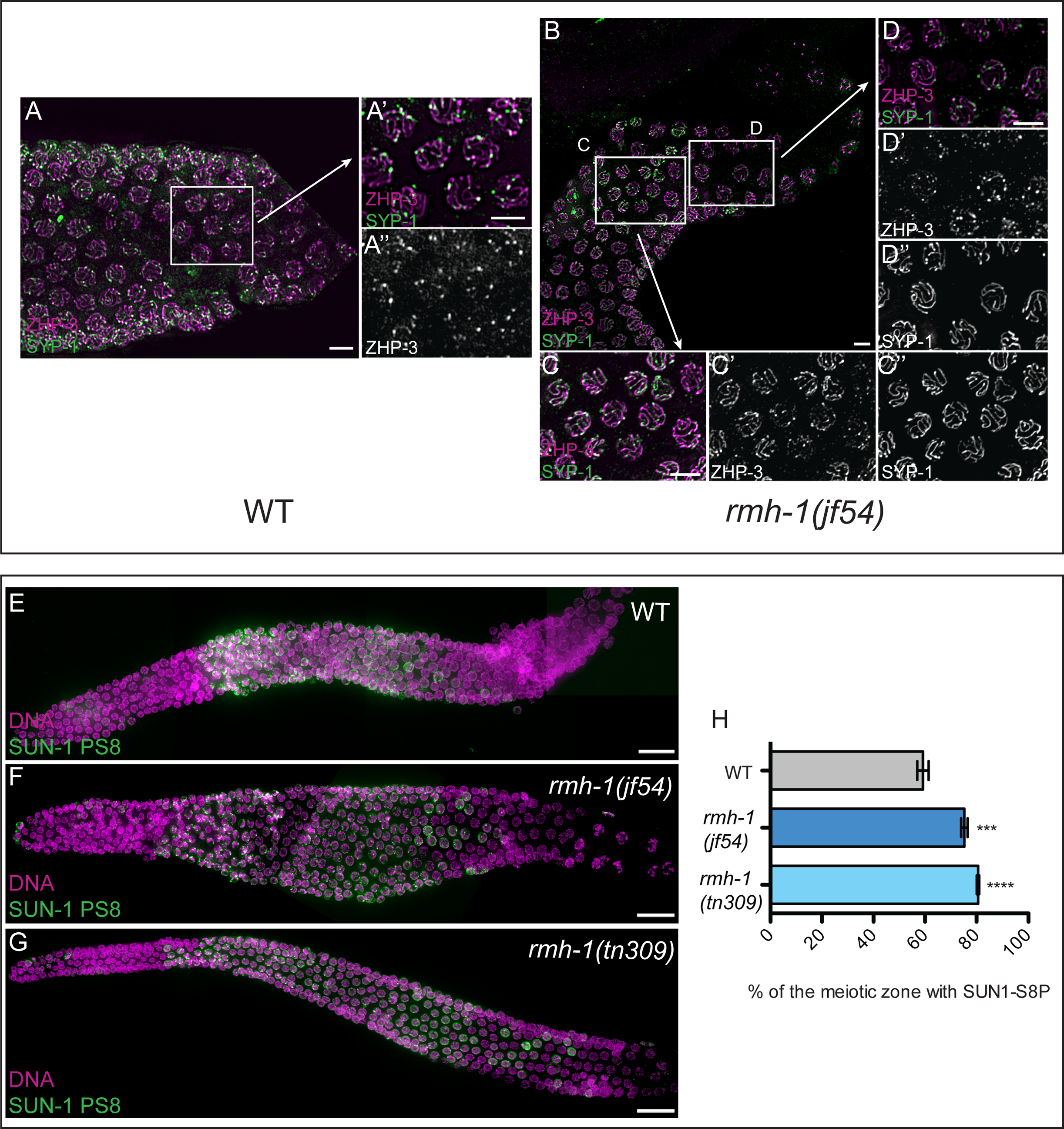

Supplement: S8 Fig — (A-D) Staining for SYP-1 and ZHP-3 on wild type (A) and rmh-1(jf54) (B). In late pachytene in wild type, ZHP-3 marks the putative CO sites (A′ and A′′), while in rmh-1(jf54), ZHP-3 is still present on the chromatin following the SC staining as stretches (D–D′′). The asymmetric retraction of SYP-1 is also delayed (C–C′′). (E–G) The SUN-1 S8 Pi positive region is extended in rmh-1. (H) Quantification of the length of the gonad zone positive for SUN-1 S8 Pi, expressed in percentage of length positive for the marker with regard to the length of zone from meiotic entry to diplotene (n = 3 gonads per genotype). Data are represented as mean +/- SD with (*** for p < 0.001 and **** for p < 0.0001). Scale bars 20 μm for gonad and 5 μm for insets. (TIF) [file pbio.1002412.s008.tif]

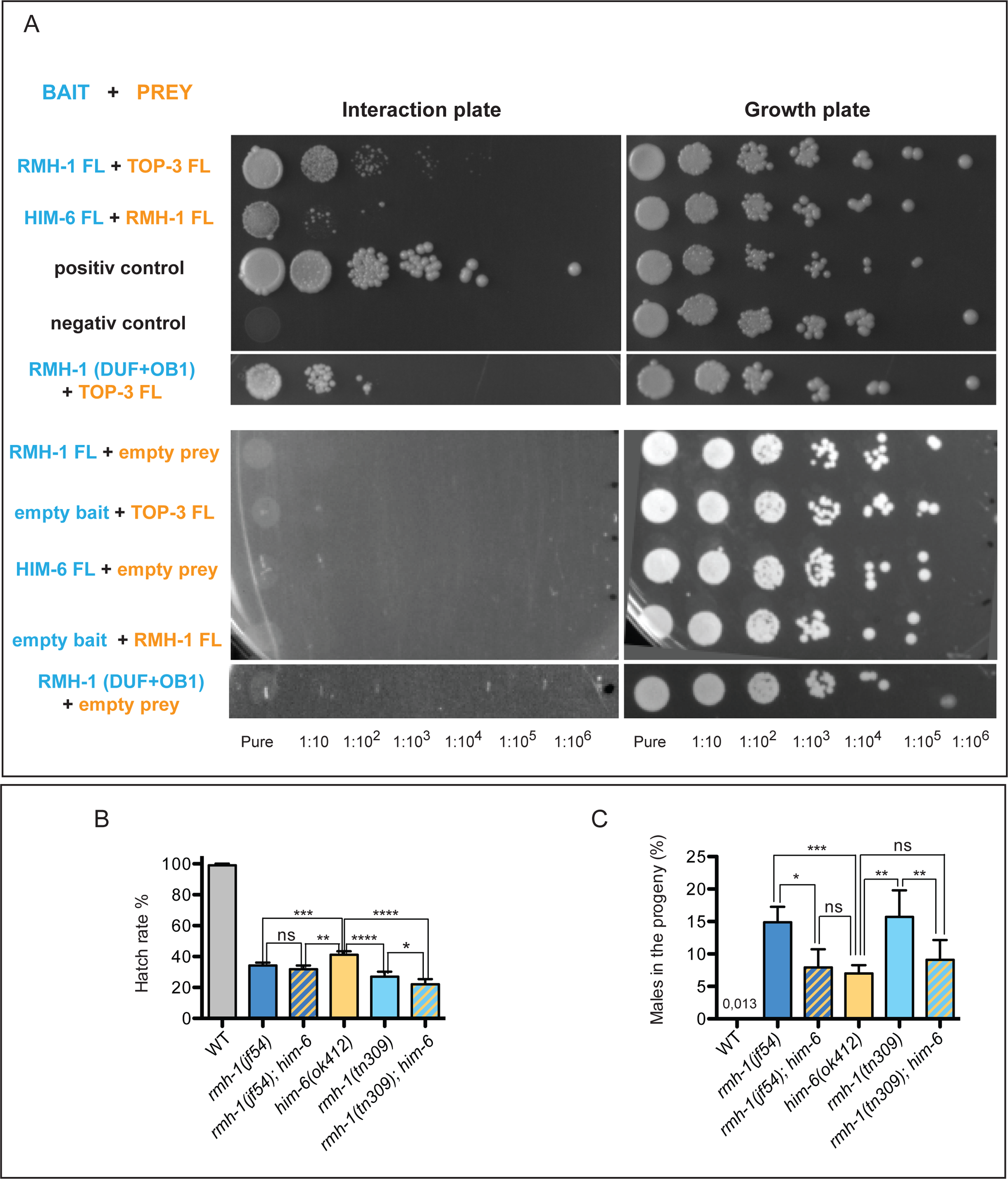

Supplement: S9 Fig — (A) Yeast two-hybrid assays for protein interactions between RMH-1, HIM-6, and TOP-3. Full-length RMH-1 interacts with TOP-3 (row 1) and with HIM-6 (row 2), respectively. A truncation protein of RMH-1 containing only the N-terminal part with the DUF and OB1 domains (reminiscent of tn309) interacts with TOP-3 (row 5). Auto-activation of both bait and prey vectors were tested in lanes 6 to 10. Interactions were scored by growth on SC-Leu-Trp-His plates (left panel) and growth on SC-Leu-Trp plates (right panel). Quantification of the percentage of hatch rate (B) and males in offspring (C) for wild type (n = 45 hermaphrodites), single mutants (rmh-1(tn309) (n = 45)—him-6(ok412) (n = 35)—rmh-1(jf54) (n = 45)), and the double mutants rmh-1(tn309); him-6 (n = 43) and rmh-1(jf54); him-6 (n = 45). Data are represented as mean +/- SD with ns (not significant) and * p < 0.05, ** p < 0.01, *** p < 0.001, **** p < 0.0001. (TIF) [file pbio.1002412.s009.tif]

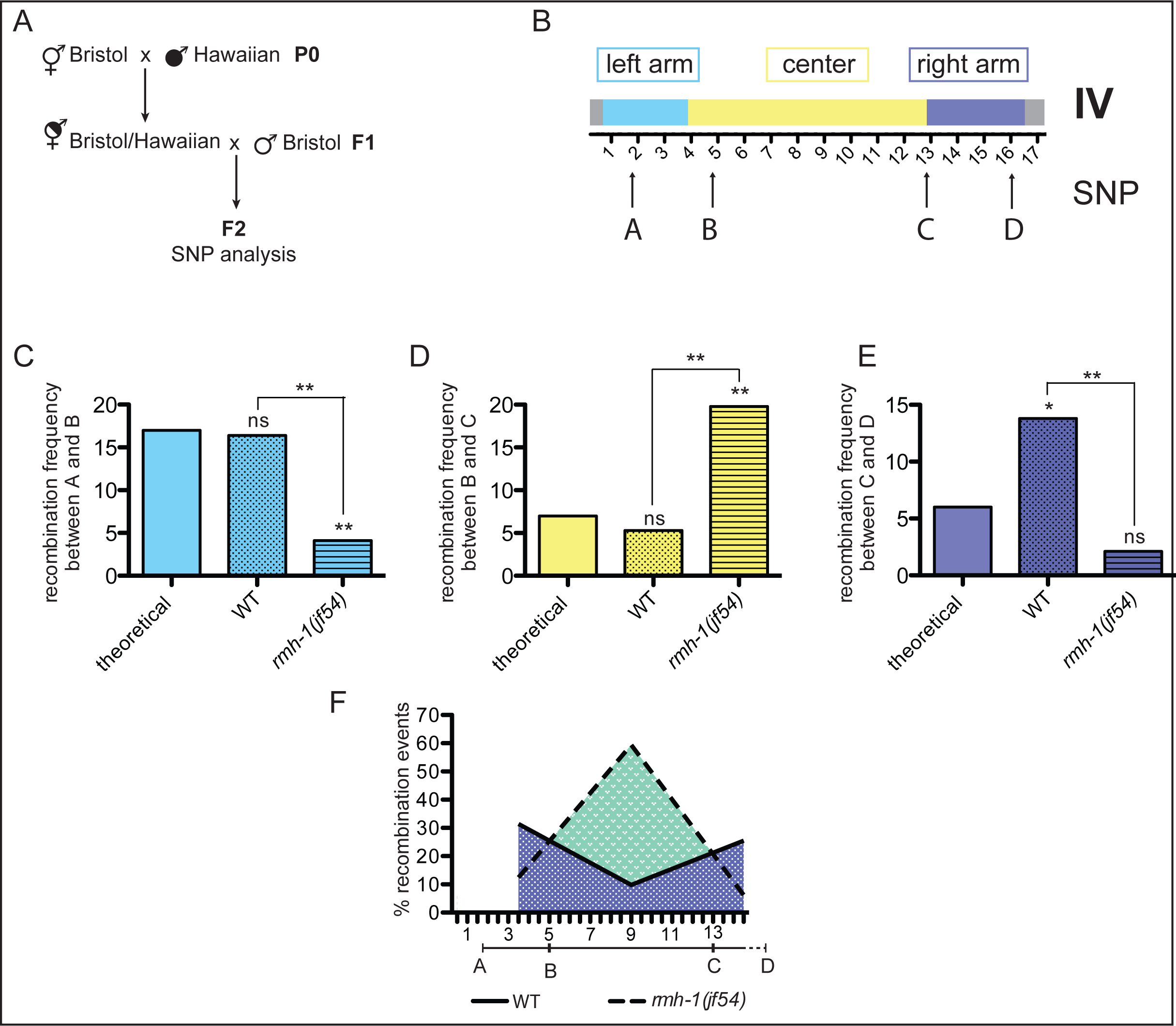

Supplement: S10 Fig — (A) Schematics of crosses to obtain the F2 individuals used for PCR-based SNP analysis. White insert indicates the wild type (Bristol) background, and black insert indicates the Hawaiian background. (B) Scheme of chromosome IV and location of the SNPs used during the recombination assay. SNP A is localized on the left arm, B is at the junction between left arm and the center, C is at the junction between the center and right arm, and D is on the right arm. (C) Recombination frequencies on chromosome IV left arm between SNP A and B for wild type (n = 94 individuals) and rmh-1(jf54) (n = 96 individuals). (D) Recombination frequencies on chromosome IV center between SNP B and C for wild type (n = 94 individuals) and rmh-1(jf54) (n = 96 individuals). COs were shifted to the centers of the chromosome in the mutant. (E) Recombination frequencies on chromosome IV right arm between SNPs C and D for wild type (n = 94 individuals) and rmh-1(jf54) (n = 96 individuals). (C–E) The column “theoretical” corresponds to the expected recombination frequency based on the published genetic distance (http://www.wormbase.org). Data are represented as percentage with ns for not significant, * for p < 0.05, and ** for p < 0.01 (Chi2 test on raw data). (F) Graph illustrating the percentage of CO events along chromosome IV for wild type and rmh-1(jf54). In the wild type, COs are concentrated to chromosome arms, whereas in the mutant, they are more concentrated at the center. (TIF) [file pbio.1002412.s010.tif]
