# Supplementary material for: Separable Roles for a Caenorhabditis elegans RMI1 Homolog in Promoting and Antagonizing Meiotic Crossovers Ensure Faithful Chromosome Inheritance
Source: PLoS Biol. 2016 Mar 24;14(3):e1002412. doi: 10.1371/journal.pbio.1002412 (PMC4807110; doi:10.1371/journal.pbio.1002412)
Supplement: S1 Text — Detailed descriptions are provided for the strains used and generated in this study, antibodies and imaging methods, FISH procedure, identification of the rmh-1 alleles, generation of rmh-1, rmh-2 alleles and him6::HA strain by CRISPR method, the tagging of RMH-1, RT-PCR procedure, measurements of DAPI bodies in diakinesis, the Y2H assay, the recombination assays by PCR-based SNP mapping, and deep sequencing. (DOCX) [file pbio.1002412.s012.docx]

# Supplemental Materials and Methods

**Worm strains**

Existing strains used: N2 (Bristol), *Hawaiian (CB4856), him-6 (ok412) IV, spo-11(me44) /nT1 V, him-18(tm2181) /hT2 III, xpf-1(tm2842) IV, mus-81(tm1937) I, slx-1(tm2644) I*, *rtel-1(tm1866) I, smc-5(tm2421) /mnl1 II, zhp-3 (jf61 ::unc-119+) /ht2 I, meIs8[unc-119(+) pie-1promoter::gfp::cosa-1] II, mIs13 [myo2p::GFP + pes10p::GFP + gut-promoter::GFP].*

The following strains were generated:

*rmh-1(jf54) I,* *rmh-1(jf92[M01E11.3 ::unc-119+]) I, rmh-1(tn309) I*, *rmh-2 (jf94[T07C12.12 ::unc-119+]) V, rmh-1(jf54) I ; rmh-2(jf94[T07C12.12 ::unc-119+]) V, rmh-1(jf54) I ; him-6 (ok412) IV, rmh-1(tn309) I ; him-6 (ok412) IV, rmh-1(jf54) I ; spo-11(me44) /nT1 V, rmh-1(jf54)/ hT2 I ; him-18(tm2181) /hT2 III, rmh-1(jf54) I ; xpf-1(tm2842) IV, rmh-1(jf54) mus-81(tm1937) /hT2 I, rmh-1(jf54) slx-1(tm2644) /hT2 I, rmh-1(jf54) rtel-1(tm1866) /hT2 I, rmh-1(jf54) I ; smc-5(tm2421) /mnl1 II, rmh-1(jf54) zhp-3(jf61 ::unc-119+)*/hT2 *I*, *zhp-3(jf61 ::unc-119+)*/ *ccls4251 unc-15(e73) I;* *smc-5(tm2421) /mnl1 II*, *rmh-1(jf54) zhp-3(jf61 ::unc-119+)*/ *ccls4251 unc-15(e73)* I; *smc-5(tm2421) /mnl1 II, jfsi38 [gfp ::rmh-1 cb-unc-119+] II, jfsi46 [mCherry ::rmh-1 cb-unc-119+] II, jfsi40 [gfp ::rmh-1(jf54) cb-unc-119+] II, rmh-1(jf92[M01E11.3 ::unc-119+]) I, jfsi40 [gfp ::rmh-1(tn309) cb-unc-119+] II, zhp-3(jf61 ::unc-119+)/hT2 I ; jfsi38 [gfp ::rmh-1 cb-unc-119+] II; jfsi38 [gfp ::rmh-1 cb-unc-119+] II; msh-5(me23)* */nT1 IV, jfsi38 [gfp ::rmh-1 cb-unc-119+] II ; cosa-1(tm3298) /Qc1 III, jfsi38 [gfp ::rmh-1 cb-unc-119+] II ; spo-11 (me44)/nT1 IV, jfsi38 [gfp ::rmh-1 cb-unc-119+] II; him-6 (ok412) IV, jfsi38 [gfp ::rmh-1 cb-unc-119+] smc-5(tm2421) /mnl1 II, him-6(jf93[him-6 ::HA] IV, jfsi38 [gfp ::rmh-1 cb-unc-119+] II; him-6(jf93[him-6 ::HA] IV, rmh-1(jf54) I ; him-6(jf93[him-6 ::HA] IV, rmh-1(tn309) I ; him-6(jf93[him-6 ::HA] IV, rmh-1(jf54) I ; meIs8[unc-119(+) pie-1promoter::gfp::cosa-1] II, rmh-1(tn309) I ; meIs8[unc-119(+) pie-1promoter::gfp::cosa-1] II, meIs8[unc-119(+) pie-1promoter::gfp::cosa-1] II; him-6 (ok412) IV, rmh-1(jf54) I ; meIs8[unc-119(+) pie-1promoter::gfp::cosa-1] II; him-6 (ok412) IV, rmh-1(jf54) I ; Hawaiian II ; Hawaiian IV; Hawaiian X , rmh-1(tn309) I ; Hawaiian IV ; Hawaiian V.*

**Fluorescence microscopy**

All microscopy evaluations were done with either a DeltaVision OMX v2 (for 3D-SIM images) or a regular Delta Vision microscope with SoftWoRx image analysis deconvolution software (Applied Precision) and ImageJ (NIH) and Adobe Photoshop software.

**Antibodies**

Primary antibodies: rabbit anti-RAD51 at 1:500 [1], rabbit anti-RAD-51 at 1:500 (Novus biological, ref 2948002) , mouse anti-GFP at 1:500 (Roche, ref 11 814 460 001), rabbit anti-MSH5 at 1:10000 (Novus biological, ref 38750002), rabbit Cy3 primary labelled mCherry at 1:200 (gift from A. Dammermann), guinea pig anti-ZHP-3 at 1:500 [2], rabbit anti-LAB-1 at 1:500 [3], guinea pig anti-SYP-1 at 1:500 [4], rabbit SYP-1 at 1:200 [5], mouse anti-PH3 (phosphorylated S10 on Histone 3) at 1:100 (Cell Signaling technology, ref 9706S), mouse anti-HA at 1:100 (Covance, Monoclonal HA. 11 clone 16B2), rabbit anti-HIM-8 at 1:10000 (Novus biological, ref 0011645), guinea pig anti-HTP-3 and rabbit anti-HTP-3 at 1:500 [6], rabbit anti-HIM-3 at 1:500 [7], guinea pig anti-phospho-SUN-1S8 (1:700) [8].

Secondary antibodies (Life technologies) were diluted at 1:500: anti-mouse Alexa488 (ref A11017), anti mouse Alexa488 (Biotum ref 20014), anti-rabbit Alexa488 (ref A11034), anti-guinea pig Alexa488 (ref A11073), anti-rabbit Alexa568 (ref A11036), anti-guinea pig Alexa568 (ref A11075), anti-guinea pig Alexa647 at 1:200 (ref A21450), anti-rabbit Alexa647 at 1:200 (ref A21245).

**Fluorescence in situ hybridization**

FISH was performed as described in [9] using a PCR generated probe against the 5 S ribosomal locus.

**Identification of *rmh-1* alleles**

The *rmh-1(jf54)* allele was recovered from an ethane methyl sulfonate (EMS) mutagenesis screen aimed at isolation of mutants with chromosome segregation defects. Males occur through the non-disjunction of the second X chromosome. Mutants with increased numbers of males were identified with the Pxol-1::GFP reporter, which is expressed specifically in males [10,11]. Independently, the recessive allele *rmh-1(tn309)* has been found in a maternal effect lethal screen [12]. SNP mapping localized the two mutations to the center of chromosome I. Subsequently genome deep sequencing revealed that both *jf54* and *tn309* alleles contained a G-to-A transition in a novel open reading frame M01E11.3. We confirmed that those two mutations were allelic with a complementation test. *trans*-heterozygote animals displayed the Him phenotype and increased DAPI bodies in diakinesis.

In the *tn309* allele, the G-to-A transition affects exon 6 by producing a premature STOP codon at position aa 640. In the *jf54* allele, the G-to-A transition affects the first nucleotide of intron 1 and therefore destroys the splice donor site of the preceding exon 1 (Fig 1A). qRT-PCR revealed the presence of different splicing variants: 24,5% of transcripts correspond to the unspliced version and 73,7% of transcripts revealed the presence of two cryptic donor sites in exon 1 (S1 B and C Fig). On one hand, the unspliced version leads to a frameshift and in consequence to the appearance of a premature STOP codon in exon 2. On the other hand, the cryptic donor sites allow the splicing of intron 1 and the produced transcripts maintain the open reading frame. However, the produced proteins contain a deletion in a functional domain of the protein (S1 Fig).

**Tagging of RMH-1**

To generate a tagged version of RMH-1, we used the Mutisite Gateway® Three-Fragment Vector Construction (Life technologies). We generated three fragments by PCR and inserted them by BP reaction into the Gateway entry clones:

1- the promoter and 5’UTR of *rmh-1* fused to the tag of interest without a STOP codon (GFP, mCherry or Dendra2) in pDONR P4-P1R

We used the following primers:

5’UTR (for): GGGGACATTGTATAGAAAAGTTGGGAAGACTACATTGAGTTGAAGC

5’UTR (rev): CTTGCTCACCATATATTTTGTAGTTTTTATATGCGTTCG

GFP (for): ACTACAAAATATATGGTGAGCAAGGGCGAGG

GFP (rev): GGGGACTGCTTTTTTGTACAAACTTGGCTTGTACAGCTCGTCCATGCC

mCherry (for) ACTACAAAATATATGGTCTCAAAGGGTGAAGAAG

mCherry (rev) GGGGACTGCTTTTTTGTACAAACTTGGTGTCGAGTGCCGCCCTTC

Dendra (for) ACTACAAAATATATGAACACCCCAGGAATCA

Dendra (rev) GGGGACTGCTTTTTTGTACAAACTTGGCCAGACTTGGGATGGGAGTGG

The 5’UTR region was fused to each tag by bridge PCR using the forward-5’UTR and reverse-tag primers.

2- coding sequence of *rmh-1* in pDONR 221:

We used the following primers:

RMH-1 (for): GGGGACAAGTTTGTACAAAAAAGCAGGCTCCATGAAAGAAACTGAACTTGATCG

RMH-1 (rev): GGGGACCACTTTGTACAAGAAAGCTGGGTTTACAAAATATTGAGAGCATCAGAA

3- the 3’UTR of *rmh-1* pDONR P2R-P3

We used the following primers:

3’UTR (for): GGGGACAGCTTTCTTGTACAAAGTGGTTATTTCTATTGCTTGCTACCTG

3’UTR (rev): GGGGACAACTTTGTATAATAAAGTTGCGATCATTAACAAAATGAACGAAT

The three fragments were combined in the destination vector pCFJ150 via the LR reaction. This last vector contains the Gateway recombination sites but also a MOS landing site (pDESTtti5605). The MosSci system allows integration of a transgene at a defined locus on chromosome II in the genome as a single copy [13].

**Strains generated by CrispR genome modifications**

We used the following plasmids: pU6 ::klp-12sgRNA (addgene 46170), peft-3 (cas9-SV40_NLS ::tbb-2 3UTR) (addgene 46168), the negative selection marker (phsp-16-41 ::peel-1::tbb-2 3UTR), the co-injections markers pCFJ90 (Pmyo-2 ::mCherry ::unc-54utr) (addgene 19327) and pGH8 (Prab-3::mCherry ::unc-54utr) (addgene 19328).

*- Guiding RNAs*

Guiding RNAs were generated by bridge PCR between the vector pU6::klp-12 sgRNA and gRNA of interest (see [14]). For the deletion strains, we used two sgRNAs surrounding the ATG for *rmh-1* and *rmh-2* ORF. For tagging HIM-6, we used only one sgRNA. The sequences of the sgRNAs we used are following:

RMH-1-sgRNA-1 : AACTTGATCGTCTTTTCTCTTGG

RMH-1-sgRNA-2 : GACACAACGAAGATTTGGCTTGG

RMH-2-sgRNA-1 : GCGCACAGACACCGCAACAT

RMH-2-sgRNA-2 : AAAATTTGCAAATCTGGTGT

HIM-6-sgRNA : AACCAGCTACTTCACTCAAACGT

*- Repair template*

For the deletion strains, the repair template was constructed as following: the *unc-119* rescue fragment was amplified by PCR from the vector pCFJ150 and cloned into pGEMteasy (Promega). Homology regions of around 1,5kb were amplified from genomic DNA and cloned before and after the *unc-119* locus.

For tagging HIM-6, we generated a PCR repair template with the mutated PAM sequence. Homology regions of around 1,5kb close to the STOP codon of the *him-6* gene were amplified from genomic DNA. The HA tag was inserted on the primer used to generate the 3’ homology arm.

*- Worm injection*

For the deletion strains, the injection mix was the following: guiding RNAs (20ng/ul each), UNC-119 repair template (25ng/ul), peft-3 at 20ng/ul, the negative selection marker at 10ng/ul and the co-injections markers pCFJ90 and pGH8 at 5ng/ul each.

For tagging HIM-6, the injection mix was the following: guiding RNA (20ng/ul), repair template (25ng/ul), peft-3 at 20ng/ul, and the co-injections markers pCFJ90 and pGH8 at 5ng/ul each and UNC-119 cloned in pGEMteasy at 20ng/ul.

*unc-119 (ed9) III* worms were injected as young adults.

*- selection*

For the deletion strains, worms were kept at 25°C until the F2 generation. *Peel-1* expression was induced by heat-shock for one hour at 34°C. After four hours, transformants were selected by rescue of the *unc* phenotype and mCherrry fluorescence. CripsR events were screened for moving worms that lost the mCherry co-injection markers. Genome modifications were identified by PCR.

For HIM-6::HA, worms were kept at 25°C until the F1 generation. Moving worms were singled and kept at 25°C. From plates transmitting an extra chromosomal array, *unc* worms were singled. Genome modifications were identified by PCR.

**Real time PCR**

Total RNA was isolated from adult hermaphrodites using TRIzol (Invitrogen, Carlsbad, CA), following the manufacturer's instructions. mRNA purification and cDNA production were performed with the Takara Primescript kit. Real time PCR was performed using Promega GoTaq@Mastermix kit. To test the expression of *rmh-1* in wild type and *rmh-1(jf54)* mutants, the following primer pairs were used:

Reverse-exon2 CGATCAGAGGCCACATCTTT

Forward-cryptic site1- junction exon1-2 CAACGAGGCA GTTATTTCCTC

Forward-cryptic site2- junction exon1-2 GAGCGCAGTAAAAGTT GTTATTTCCTC

Forward- WT junction exon1-2 GAATGATTTTCCAG GTTATTTCCTC

Forward- Intron 1 ATCTGCCGAAAATCAACAGG

**Irradiation**

Hermaphrodites were exposed to ionizing irradiation from a 137Cs source (50 Gy). Dissection and cytological analysis were done 4h or 8h post-irradiation.

**Measurement of the volume of DAPI bodies in diakinesis**

We adapted the method used in [15] to detected fragmentation in the *smc-3* mutant. We measured the volume of DAPI bodies using the 3D Object Counter plugin of Image J. As a source of images, we used our deconvolved stack images. Classes were defined by the volume of the DAPI structures in the last diakinesis oocyte prior fertilization. Using this method, we established that a bivalent exhibits an average volume of 3.3 ± 1.3 μm^3^ (observed in the wild type) and a univalent exhibits an average volume of 1.4 ± 0.6 μm^3^ (observed in a *msh-5* mutant defective for CO formation thus producing only univalents). DAPI structures with a larger volume than a univalent were classified as “DNA masses” and DAPI structures with a smaller volume of a univalent were classified as fragments.

**Yeast two hybrid**

Yeast two hybrid assays were conducted after [16]. cDNA of RMH-1 full length, RMH-1 truncation^11–216^, HIM-6, TOP-3 were cloned into the three following vectors into the SacII and XmaJI sites: the prey vector pDP133 and the two bait vectors pDP134 where the LexA tag is C-terminal and pDP135 where the LexA tag is N-terminal. The yeast strain Mat-a was cotransfected with pray and bait vector and were selected on SC -Leu -Trp plates. Interaction was assayed on SC -Leu -Trp - His plates after two to four days of growth at 30°C. Growth was assayed on SC -Leu -Trp plates after two to four days of growth at 30°C.

**Meiotic recombination assay with PCR based SNP analysis**

We assayed recombination frequencies and CO localisation by using SNPs that differ between N2 Bristol (WT) and Hawaiian (HA) (four SNPs for chromosome IV). *rmh-1(jf54)* was crossed to the HA strain to generate a mutant with introgressed HA chromosome IV. Genetic crosses were done as described in S10 Fig. *rmh-1* mutants males in HA and *rmh-1* mutants hermaphrodites in WT were crossed to generate F1 *rmh-1* mutants worms heterozygotes for HA on chromosomes IV. Recombination events were allowed to take place in F1 worms. Those were mated with WT expressing a pharyngeal GFP transgene to introduce a WT paternal chromosome and allow the exclusive monitoring of the recombination event in oogenesis. After laying eggs, F1 mums were lysed and then genotyped to ensure that the first mating worked using the presence of Hawaiian SNPs as a read-out. Singled F2 hermaphrodites were lysed and subjected to PCR:

Primers used for chromosome IV (restriction with DraI) adapted from [17]:

SNP A (position – 16): CGCATAAATCCAACGTTCTCTG and AATCCATAAGTTTCGTGTTGG

SNP B (position 1): AAAATGGGAAGCGTACCAAA and TGCTTGTAGCGTTTCCAAGA

SNP C (position 8): GACACGACTTTAGAAACAACA and TGGTATGGAGTCCCTATTTTG

SNP D (position 14): GAATTTCAGGTGTTGGAAGG and TGCTCTGAAAAAATTGGCTG

In S10 Fig, CO positions were classified as described in [18]: left arm, center and right arm of chromosomes.

**Meiotic recombination assay by deep sequencing**

We also crossed *rmh-1(tn309)* to the Hawaiian strain to generate a mutant with several introgressed HA chromosomes (chromosomes X, IV and V). The crossing scheme is identical to the PCR SNP based recombination assay. F2 GFP positives worms were singled and allowed to grow to starvation, harvested with PBS and genomic DNA prepared using the GentraPure kit of Qiagen (50µl of frozen worm pellet).

DNA sample were then sheared using a S220 ultrasonicator (Covaris). DNA library was prepared using a kit from New Englands Biolabs. Samples were then sequenced on an Illumina HiSeq2500 with 125 PE-sequencing. Samples preparation and sequencing was performed at the CSF NGS unit of Vienna (www.csf.ac.at).

Bioinformatic analysis of the results was performed as following:

*- Mapping*

Paired-end reads were trimmed with trimmomatic [19] to eliminate low quality reads and adapter contamination (default parameters). The remaining reads were mapped to the *C. elegans* WBcel235 reference genome (release 79; Ensembl database) using NextGenMap version 0.4.12 [20] with default parameters. Subsequently samtools and picard ([21], Broad Institute) were used to remove unmapped reads (SAM flag 4), pairs with unmapped mate (SAM flag 8), reads with low mapping quality (Q<20) and duplicated reads (picard MarkDuplicates). Once having high quality reads, the coverage was estimated and all samples with coverage below 5x were discarded.

*- SNP calling*

SNP calling was done with vcftools [22], using parameters -min-meanDP 5 -max-meanDP 200 -minQ 10. For the two parental strains, Laboratory Bristol (Br) and Hawaiian (Hw), all the variants found when comparing to the reference genome were classified as follows: heterozygous unique if it was only found in one of the parental strains but also calling the reference, heterozygous shared if it was found in both parental strains but also calling the reference, heterozygous unshared if each of the parental had a different call and also calling the reference, homozygous unique if it was only found in one of the parental strains fixed, homozygous shared if it was found in both parental strains fixed, homozygous unshared if each of the parental had a different call fixed, and polymorphic if more than two nucleotides were called.

| SNPs type | Laboratory/Bristol | Hawaiian |
| --- | --- | --- |
| Heterozygous unique | 1,694 | 57,524 |
| Heterozygous shared | 331 | 233 |
| Heterozygous unshared | 0 | 0 |
| Homozygous unique | 326 | 166,928 |
| Homozygous shared | 239 | 337 |
| Homozygous unshared | 0 | 0 |
| Total | 2,590 | 225,022 |
| Total Polymorphic | 2.8% | 2.7% |

Finally to avoid having two or more SNPs in the same read pair, they were selected to be at least 400 bp apart from each other starting from the beginning of the chromosome. To be able to assess the chromosome composition in each of the candidates, we only used homozygous unique SNPs from the Hw strain, as the number of SNPs from the Br Strain were negligible. For the 72 candidates, only the information corresponding to the homozygous unique SNPs from the Hw stain was kept and new variants were ignored.

| Chromosome | Number  of SNPs | Number of SNPs per bin and bin size | | | |
| --- | --- | --- | --- | --- | --- |
|  |  | 10,000 bins | 5,000 bins | 2,000 bins | 1,000 bins |
| I | 9,539 | 0 – 4 in 1.5kb | 0 – 7 in 3kb | 0 – 19 in 7.5kb | 0 – 38 in 15kb |
| II | 10,841 | 0 – 4 in 1.5kb | 0 – 8 in 3kb | 0 – 19 in 7.6kb | 0 – 38 in 15kb |
| III | 7,826 | 0 – 3 in 1.4kb | 0 – 7 in 2.8kb | 0 – 17 in 6.9kb | 0 – 34 in 14kb |
| IV | 9,438 | 0 – 4 in 1.7kb | 0 – 9 in 3.5kb | 0 – 22 in 8.7kb | 0 – 44 in 17kb |
| V | 14,895 | 0 – 5 in 2.1kb | 0 – 10 in 4.2kb | 0 – 26 in 10.4kb | 0 – 52 in 21kb |
| X | 8,848 | 0 – 4 in 1.8kb | 0 – 9 in 3.5kb | 0 – 22 in 8.9kb | 0 – 44 in 18kb |
| Total | 61,387 |  |  |  |  |

*- Detection of breakpoints in the recombinant candidates*

In the absence of recombination two genotypes are expected in the offspring: fully homozygous (i.e., both homologous chromosomes come from the same parental strain) and fully heterozygous (each homolog come from one parental strain). If recombination occurred blocks of homozygosity and blocks of heterozygosity will co-occur on homologous chromosomes. We identified the recombination break-points by analyzing the changes in heterozygosity along the chromosome.

As we had a high variability in the coverage of the samples, we used a binning method to increase the power of the test in the sites of interest. We split the chromosome into *n* (1,000, 2,000, 5,000 or 10,000) bins such as the minimum number of reads that cover the SNPs of interest in each bin is 50x. Then for each bin we pooled the number of reads calling Bristol or Hawaiian SNP-alleles and compute a binomial test (p of success of Bristol in heterozygous 0.6) then called that bin heterozygous if the p-values > 0.05 or homozygous if p-values <= 0.05. Finally we scanned the whole chromosome looking for bins that switch from homozygosity to heterozygosity, heterozygosity to homozygosity or Bristol homozygous to Hawaiian homozygous. If we observe in two consecutive bins a switch the corresponding genomic region is called putative recombination break-point (PRB). To further corroborate this we require that 75% of the bins of at least one side of the PRB do not switch, if we consider 10% of all bins centered at the PBR.

| Candidates | Number  of samples | Number of recombination events | Average observed events |
| --- | --- | --- | --- |
| Control | 15 | 37 | 2.4 per sample |
| tn309 | 14 | 51 | 3.6 per sample |

# All the sequences obtained by deep sequencing have been uploaded on the NCBI public database under the project PRJNA298297.

# Supplemental References

1. Colaiácovo MP, MacQueen AJ, Martinez-Perez E, McDonald K, Adamo A, La Volpe A, et al. Synaptonemal complex assembly in C. elegans is dispensable for loading strand-exchange proteins but critical for proper completion of recombination. Dev Cell. 2003;5: 463–474.

2. Bhalla N, Wynne DJ, Jantsch V, Dernburg AF. ZHP-3 acts at crossovers to couple meiotic recombination with synaptonemal complex disassembly and bivalent formation in C. elegans. PLoS Genet. 2008;4: e1000235. doi:10.1371/journal.pgen.1000235

3. de Carvalho CE, Zaaijer S, Smolikov S, Gu Y, Schumacher JM, Colaiácovo MP. LAB-1 antagonizes the Aurora B kinase in C. elegans. Genes Dev. 2008;22: 2869–2885. doi:10.1101/gad.1691208

4. Nabeshima K, Villeneuve AM, Colaiácovo MP. Crossing over is coupled to late meiotic prophase bivalent differentiation through asymmetric disassembly of the SC. J Cell Biol. 2005;168: 683–689. doi:10.1083/jcb.200410144

5. MacQueen AJ, Colaiácovo MP, McDonald K, Villeneuve AM. Synapsis-dependent and -independent mechanisms stabilize homolog pairing during meiotic prophase in C. elegans. Genes Dev. 2002;16: 2428–2442. doi:10.1101/gad.1011602

6. Goodyer W, Kaitna S, Couteau F, Ward JD, Boulton SJ, Zetka M. HTP-3 links DSB formation with homolog pairing and crossing over during C. elegans meiosis. Dev Cell. 2008;14: 263–274. doi:10.1016/j.devcel.2007.11.016

7. Zetka MC, Kawasaki I, Strome S, Müller F. Synapsis and chiasma formation in Caenorhabditis elegans require HIM-3, a meiotic chromosome core component that functions in chromosome segregation. Genes Dev. 1999;13: 2258–2270.

8. Penkner AM, Fridkin A, Gloggnitzer J, Baudrimont A, Machacek T, Woglar A, et al. Meiotic Chromosome Homology Search Involves Modifications of the Nuclear Envelope Protein Matefin/SUN-1. Cell. 2009;139: 920–933. doi:10.1016/j.cell.2009.10.045

9. Penkner A, Portik-Dobos Z, Tang L, Schnabel R, Novatchkova M, Jantsch V, et al. A conserved function for a Caenorhabditis elegans Com1/Sae2/CtIP protein homolog in meiotic recombination. EMBO J. 2007;26: 5071–5082. doi:10.1038/sj.emboj.7601916

10. Kelly KO, Dernburg AF, Stanfield GM, Villeneuve AM. Caenorhabditis elegans msh-5 is required for both normal and radiation-induced meiotic crossing over but not for completion of meiosis. Genetics. 2000;156: 617–630.

11. Tang L, Machacek T, Mamnun YM, Penkner A, Gloggnitzer J, Wegrostek C, et al. Mutations in Caenorhabditis elegans him-19 show meiotic defects that worsen with age. Mol Biol Cell. 2010;21: 885–896. doi:10.1091/mbc.E09-09-0811

12. Furuta T, Tuck S, Kirchner J, Koch B, Auty R, Kitagawa R, et al. EMB-30: an APC4 homologue required for metaphase-to-anaphase transitions during meiosis and mitosis in Caenorhabditis elegans. Mol Biol Cell. 2000;11: 1401–1419.

13. Frøkjaer-Jensen C, Wayne Davis M, Hopkins CE, Newman BJ, Thummel JM, Olesen S-P, et al. Single-copy insertion of transgenes in Caenorhabditis elegans. Nat Genet. 2008;40: 1375–1383. doi:10.1038/ng.248

14. Friedland AE, Tzur YB, Esvelt KM, Colaiácovo MP, Church GM, Calarco JA. Heritable genome editing in C. elegans via a CRISPR-Cas9 system. Nat Meth. 2013;10: 741–743. doi:10.1038/nmeth.2532

15. Baudrimont A, Penkner A, Woglar A, Mamnun YM, Hulek M, Struck C, et al. A New Thermosensitive smc-3 Allele Reveals Involvement of Cohesin in Homologous Recombination in C. elegans. PLoS ONE. 2011;6: e24799. doi:10.1371/journal.pone.0024799

16. Kraft C, Kijanska M, Kalie E, Siergiejuk E, Lee SS, Semplicio G, et al. Binding of the Atg1/ULK1 kinase to the ubiquitin-like protein Atg8 regulates autophagy. EMBO J. 2012;31: 3691–3703. doi:10.1038/emboj.2012.225

17. Rosu S, Libuda DE, Villeneuve AM. Robust Crossover Assurance and Regulated Interhomolog Access Maintain Meiotic Crossover Number. Science. 2011;334: 1286–1289. doi:10.1126/science.1212424

18. Rockman MV, Kruglyak L. Recombinational landscape and population genomics of Caenorhabditis elegans. PLoS Genet. 2009;5: e1000419. doi:10.1371/journal.pgen.1000419

19. Bolger AM, Lohse M, Usadel B. Trimmomatic: a flexible trimmer for Illumina sequence data. Bioinformatics. 2014;30: 2114–2120. doi:10.1093/bioinformatics/btu170

20. Sedlazeck FJ, Rescheneder P, Haeseler von A. NextGenMap: fast and accurate read mapping in highly polymorphic genomes. Bioinformatics. 2013;29: 2790–2791. doi:10.1093/bioinformatics/btt468

21. Li H, Handsaker B, Wysoker A, Fennell T, Ruan J, Homer N, et al. The Sequence Alignment/Map format and SAMtools. Bioinformatics. 2009;25: 2078–2079. doi:10.1093/bioinformatics/btp352

22. Danecek P, Auton A, Abecasis G, Albers CA, Banks E, DePristo MA, et al. The variant call format and VCFtools. Bioinformatics. 2011;27: 2156–2158. doi:10.1093/bioinformatics/btr330
